# The influence of electronic reminders on recording diagnoses in a primary health care emergency department: a register-based study in a Finnish town

**DOI:** 10.1080/02813432.2021.1910449

**Published:** 2021-04-14

**Authors:** Mika Lehto, Kaisu Pitkälä, Ossi Rahkonen, Merja K. Laine, Marko Raina, Timo Kauppila

**Affiliations:** a Department of General Practice and Primary Health Care, University of Helsinki and Helsinki University Hospital, Helsinki, Finland; b Vantaa Health Centre, City of Vantaa, Finland; c Department of Public Health, University of Helsinki, Helsinki, Finland; d Folkhälsan Research Centre, Helsinki, Finland

**Keywords:** Diagnose, emergency department, electronic medical health record, electronic reminder, primary care, recording

## Abstract

**Objective:**

This study examines whether implementation of electronic reminders is associated with a change in the amount and content of diagnostic data recorded in primary health care emergency departments (ED).

**Design:**

A register-based 12-year follow-up study with a before-and-after design.

**Setting:**

This study was performed in a primary health care ED in Finland. An electronic reminder was installed in the health record system to remind physicians to include the diagnosis code of the visit to the health record.

**Subjects and main outcome measures:**

The report generator of the electronic health record-system provided monthly figures for the number of different recorded diagnoses by using the International Classification of Diagnoses (ICD-10th edition) and the total number of ED physician visits, thus allowing the calculation of the recording rate of diagnoses on a monthly basis and the comparison of diagnoses before and after implementing electronic reminders.

**Results:**

The most commonly recorded diagnoses in the ED were acute upper respiratory infections of various and unspecified sites (5.8%), abdominal and pelvic pain (4.8%), suppurative and unspecified otitis media (4.5%) and dorsalgia (4.0%). The diagnosis recording rate in the ED doubled from 41.2 to 86.3% (*p* < 0.001) after the application of electronic reminders. The intervention especially enhanced the recording rate of symptomatic diagnoses (ICD-10 group-R) and alcohol abuse-related diagnoses (ICD-10 code F10). Mental and behavioural disorders (group F) and injuries (groups S-Y) were also better recorded after this intervention.

**Conclusion:**

Electronic reminders may alter the documentation habits of physicians and recording of clinical data, such as diagnoses, in the EDs. This may be of use when planning resource managing in EDs and planning their actions.

**KEY POINTS**
- Electronic reminders enhance recording of diagnoses in primary care but what happens in emergency departments (EDs) is not known.
- Electronic reminders enhance recording of diagnoses in primary care ED.
- Especially recording of symptomatic diagnoses and alcohol abuse-related diagnoses increased.

## Introduction

Electronic reminders have been reported to have some effects when modifying the clinical practice of physicians in primary care [1]. In order to alter physicians’ clinical habits in emergency departments (EDs), electronic reminders have been used for decreasing inappropriate antibiotic [2] and opioid prescription [3]. They have been reported to be useful in promoting the use of an HIV screening program [4] and in enhancing adherence to HIV treatment guidelines [5]. In order to enhance preventive work in EDs, electronic reminders s have been introduced to promote the administration of pneumococci vaccination to patients meeting predetermined criteria for its use [6]. Not all interventions with ERs have been successful, or their impact has been marginal (reviewed in [7]).

One important function of electronic reminders is the improvement of the quality of documentation in EDs [8]. Having recently observed that electronic reminders are effective in increasing the recording rate of diagnoses in ordinary visits of primary care physicians [9,10], it is important to study the association of electronic reminders with documentation in an ED setting. We studied changes in the diagnosis recording rate in a primary health care ED which provides care for unscreened emergency patients in its service area [11,12]. The primary aim of this study was to evaluate whether implementation of electronic reminders altered the rate of recording diagnoses or the content of diagnostic data recorded in a primary health care ED setting.

## Methods

### Setting and design

The present work is a register-based longitudinal follow-up study with a before-and-after design in the primary care of the city of Vantaa, the fourth largest city of Finland, having about 200,000 inhabitants in the year 2008. This study was performed in the primary health care ED (described in detail earlier [11,12]) which treated all those patients entering the ED without direct referral to specialist care. The physicians working in the ED were both General Practitioners (GPs) and unspecialized primary care physicians. A proportion of them worked permanently in the ED whereas some of them were regular GPs doing occasional duty. The specialist health care ED (Helsinki University Hospital, HUS, Helsingin ja Uudenmaan sairaanhoitopiiri) was located adjacent to the primary care ED and in the case of a need of specialist care the patient was referred to the secondary care [11,12]. The Finnish primary health care and its electronic health record -systems are maintained by municipalities and funded mostly with tax income.

#### Ethics

This study was carried out by examining data from the electronic health record-system without identifying the patients or ED physicians. The register holder (the health authorities of Vantaa) and the scientific ethical board of Vantaa City (TUTKE) granted permission (VD/8059/13.00.00/2016) to carry out the study.

#### Data acquisition

The data of the Vantaa primary health care ED system were obtained from the Graphic Finstar-electronic health record system (GFS, Logica LTD, Helsinki, Finland). GFS provides a specific field in the electronic health record-system where an appropriate diagnosis code (based on the 10th version of the International Classification of Diseases, ICD-10) could be entered during the patient’s visit to the ED physician. The system assists the physician in assigning an appropriate diagnosis code or allows the physician to enter the desired diagnosis code to the system directly as described in detail earlier [9,10]. The GFS system prompted ED physicians to enter a diagnosis code every time they wanted to complete recording the visit [9,10]. Upon encounter completion, the electronic health record system prompted the physician of the missing diagnosis code with an additional pop-up question ‘Are you sure you wish to complete the recording without including a diagnosis code?’. The physician then had a possibility to continue completing the recording by answering ‘Yes’ or alternatively, return to the encounter by answering ‘No’ and including the diagnosis code before eventually closing the encounter. The ED had no financial incentives associated with diagnose coding.

### Primary and secondary measures

The report generator of the GFS system provided monthly figures for the number of different recorded diagnoses and the total number of ED physician visits, thus allowing the calculation of the recording rate of diagnoses on a monthly basis without identifying individual ED physicians or patients. For analysis, the ICD-10 diagnoses were collected and examined at accuracies of three digits and initial letters. Distributions of the diagnoses recorded in the ED were used as a measure for analysis in this study. The twenty most commonly recorded diagnoses were analyzed in more detail. In addition, the proportion of the visits having a recorded diagnosis in the ED was investigated.

The whole follow-up period consists 6-year time-period before the installation of the electronic reminder into the GFS. This intervention took place on February 1st, 2008. The data was available until December 31st, 2014. After that the ED was outsourced to HUS. Thus, the follow-up lasted altogether for 12 years. The obtained data were analyzed by comparing the rates and proportions of the 20 most frequently recorded diagnoses during the six-year time periods before (2002–2007) and after (2009–2014) the year of the installation of the electronic reminder into the electronic health record system (2008).

### Statistical analyses

The comparisons of percentages or amounts of diagnoses before (2002–2007) and after (2009–2014) implementation of the electronic reminder were performed with *t*-test, Mann-Whitney *U* test or Χ^2^ test when appropriate. The rate of change in diagnosis recording was analyzed by using a general linear model of regression analysis allowing us to detect the mean change in the rate of recorded diagnoses (%/month) and its standard error of the mean (SEM) before, at the beginning of the intervention and at the stable state of the intervention (GLM procedure of SigmaPlot 10.0 Statistical Software, Systat Software Inc., Richmond, CA). These rates were then compared with *t*-test [13–15], and *p* < 0.05 was considered to indicate a statistically significant difference.

## Results

### Distribution of diagnoses

During the whole follow-up period, there was a total of 605,704 visits to the ED. Diagnoses were recorded for 350,134 (58%) of these visits. In the ED, visits having one of the 20 most commonly recorded diagnoses constituted 45.9% of the visits for which a diagnosis was recorded (Table 1), and 26.5% of all recorded visits. Altogether, 1310 different diagnoses were assigned to the patients. The most commonly recorded diagnoses in the ED were acute upper respiratory infections (5.8%), gastric or pelvic pain (4.8%), middle ear infection (4.5%), back pain (4%), wound in head (2.7%) and acute bronchitis (2.7%) (Table 1).

**Table 1.** Cumulative percentage of visits to the primary health care emergency department physicians as a function of different recorded 10th edition International Classification of Diseases (ICD-10) diagnoses in the city of Vantaa, Finland.

Diagnosis	ICD-10 code	*N*	%	Cumulative %	Diagnosis	ICD-10 code	*N*	%	Cumulative %
Acute upper respiratory infections of multiple and unspecified sites	J06	20381	5.82	5.82	Syncope and collapse	R55	1549	0,44	66.70
Abdominal and pelvic pain	R10	16843	4.81	10.63	Pain, not elsewhere classified	R52	1543	0,44	67.14
Suppurative and unspecified otitis media	H66	15717	4.49	15.12	Otitis externa	H60	1532	0,44	67.58
Dorsalgia	M54	13845	3.95	19.07	Haemorrhage from respiratory passages	R04	1526	0,44	68.02
Open wound of head	S01	9401	2.68	21.76	Convulsions, not elsewhere classified	R56	1500	0,43	68.44
Acute bronchitis	J20	9306	2.66	24.42	Urticaria	L50	1497	0,43	68.87
Other gastroenteritis and colitis of infectious and unspecified origin	A09	6917	1.98	26.39	Shoulder lesions	M75	1462	0,42	69.29
Mental and behavioural disorder due to use of alcohol	F10	6879	1.96	28.36	Acute tubulo-interstitial nephritis	N10	1457	0,42	69.71
Pain in throat and chest	R07	6520	1.86	30.22	Dislocation, sprain and strain of joints and ligaments of knee	S83	1436	0,41	70.12
Dislocation, sprain and strain of joints and ligaments at ankle and foot level	S93	6160	1.76	31.98	Dislocation, sprain and strain of joints and ligaments of shoulder girdle	S43	1430	0,41	70.52
Open wound of wrist and hand	S61	5927	1.69	33.67	Viral and other specified intestinal infections	A08	1401	0,40	70.92
Cystitis	N30	5919	1.69	35.36	Cellulitis	L03	1398	0,40	71.32
Acute tonsillitis	J03	5785	1.65	37.01	Other dorsopathies, not elsewhere classified	M53	1393	0,40	71.72
Conjunctivitis	H10	5499	1.57	38.58	Atopic dermatitis	S20	1371	0,39	72.11
Acute sinusitis	J01	5375	1.54	40.12	Other headache syndromes	G44	1358	0,39	72.50
Other soft tissue disorders, not elsewhere classified	M79	4508	1.29	41.41	Atrial fibrillation and flutter	I48	1260	0,36	72.86
Intracranial injury	S06	4240	1.21	42.62	Superficial injury of lower leg	S81	1206	0,34	73.20
Malaise and fatigue	R53	4078	1.16	43.78	Problems related to lifestyle	Z72	1160	0,33	73.54
Abnormalities of breathing	R06	3694	1.06	44.84	Other functional intestinal disorders	K59	1147	0,33	73.86
Fracture of forearm	S52	3672	1.05	45.89	Disorders of vestibular function	H81	1135	0,32	74.19
Nonsuppurative otitis media	H65	3605	1.03	46.92	Fracture of rib(s), sternum and thoracic spine	S22	1085	0,31	74.50
Dizziness and giddiness	R42	3516	1.00	47.92	Foreign body on external eye	T15	1066	0,30	74.80
Urinary tract infection, site not specified	N39	3475	0.99	48.91	Heart failure	I50	996	0,28	75.09
Pneumonia, organism unspecified	J18	3361	0.96	49.87	Angina pectoris	I20	992	0,28	75.37
Headache	R51	3221	0.92	50.79	Epilepsy	G40	980	0,28	75.65
Depressive episode	F32	3062	0.87	51.67	Schizophrenia	F20	939	0,27	75.92
Fracture at wrist and hand level	S62	3055	0.87	52.54	Open wound of ankle and foot	S91	936	0,27	76.19
Maltreatment syndromes	T74	2888	0.82	53.36	Dyspepsia	K30	923	0,26	76.45
Superficial injury of wrist and hand	S60	2768	0.79	54.16	Tachycardia, unspecified	R00	914	0,26	76.71
Acute laryngitis and tracheitis	J04	2760	0.79	54.94	Reaction to severe stress and adjustment disorders	F43	913	0,26	76.97
Erysipelas	A46	2669	0.76	55.71	Calculus of kidney and ureter	N20	893	0,26	77.23
Acute pharyngitis	J02	2630	0.75	56.46	Internal derangement of knee	M23	889	0,25	77.48
Fever of other and unknown origin	R50	2615	0.75	57.20	Superficial injury of forearm	S50	851	0,24	77.72
Other anxiety disorders	F41	2605	0.74	57.95	Phlebitis and thrombophlebitis	I80	843	0,24	77.96
Dislocation of finger	S63	2292	0.65	58.60	Open wound of forearm	S51	813	0,23	78.20
Fracture of lower leg, including ankle	S82	2193	0.63	59.23	Delirium, not induced by alcohol and other psychoactive substances	F05	811	0,23	78.43
Asthma	J45	2024	0.58	59.81	Superficial injury of abdomen, lower back and pelvis	S30	799	0,23	78.66
Fracture of shoulder and upper arm	S42	2009	0.57	60.38	Otalgia and effusion of ear	H92	790	0,23	78.88
Migraine	G43	1919	0.55	60.93	Other disorders of fluid, electrolyte and acid-base balance	E87	767	0,22	79.10
Superficial injury of lower leg	S80	1876	0.54	61.46	Oedema, not elsewhere classified	R60	751	0,21	79.31
Nausea and vomiting	R11	1816	0.52	61.98	Diverticular disease of small intestine with perforation and abscess	K57	742	0,21	79.53
Adverse effects, not elsewhere classified	T78	1795	0.51	62.50	Chronic obstructive pulmonary disease with acute lower respiratory infection	J44	718	0,21	79.73
Superficial injury of ankle and foo	S90	1770	0.51	63.00	Keratitis	H16	692	0,20	79.93
Fracture of foot, except ankle	S92	1740	0.50	63.50	Injury of Achilles tendon	S86	686	0,20	80.13
Other cardiac arrhythmias	I49	1676	0.48	63.98	Dislocation, sprain, and strain of joints and ligaments at neck level	S13	684	0,20	80.32
Essential (primary) hypertension	I10	1668	0.48	64.45	Cholelithiasis	K80	669	0,19	80.51
Superficial injury of head	S00	1594	0.46	64.91	Other enthesopathies	M77	665	0,19	80.70
Cutaneous abscess, furuncle and carbuncle of face	L02	1586	0.45	65.36	Haemorrhoids	I84	662	0,19	80.89
Influenza with pneumonia, virus not identified	J11	1583	0.45	65.81	Bacterial pneumonia, not elsewhere classified	J15	662	0,19	81.08
Cough	R05	1559	0.45	66.26	Superficial injury of shoulder and upper arm	S40	658	0,19	81.27

One hundred most common diagnoses are shown. For total data see Supplementary table.

### Association between electronic reminders and frequency of recording diagnoses

**Figure 1.**
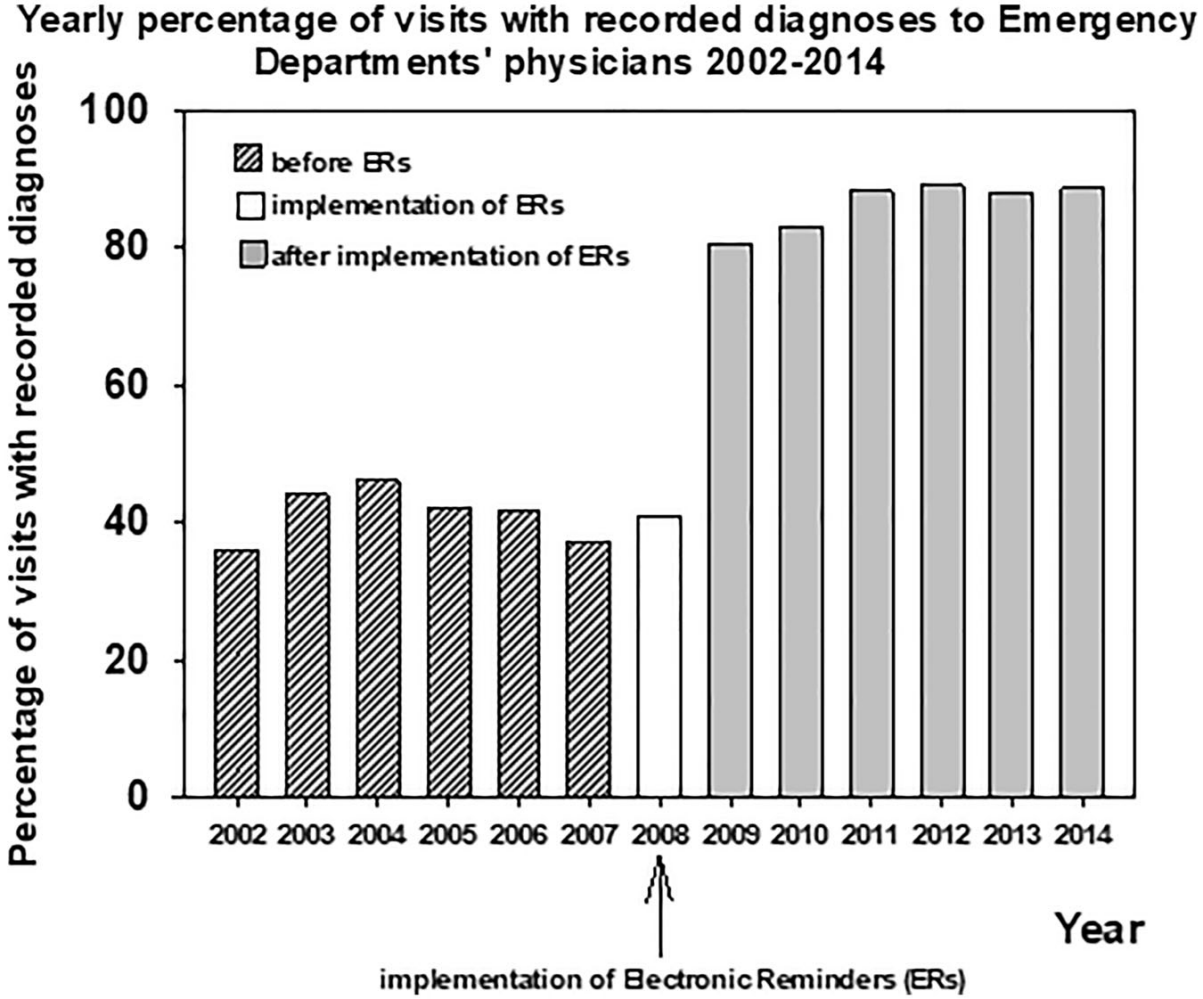
Yearly percentage of visits with recorded diagnoses to the physicians of the primary health care Emergency Department before and after implementation of the electronic reminders 2002–2014 in the city of Vantaa, Finland.

The percentage of recorded diagnoses in the ED increased by 109% after the application of electronic reminders (Figure 1). The diagnosis recording rate for visits to ED physicians increased from 41.3 (SD 3.9, SD) (first 6 years before intervention) to 86.3 (SD 3.5) (last 6 years of the intervention, *p* < 0.001).

**Figure 2.**
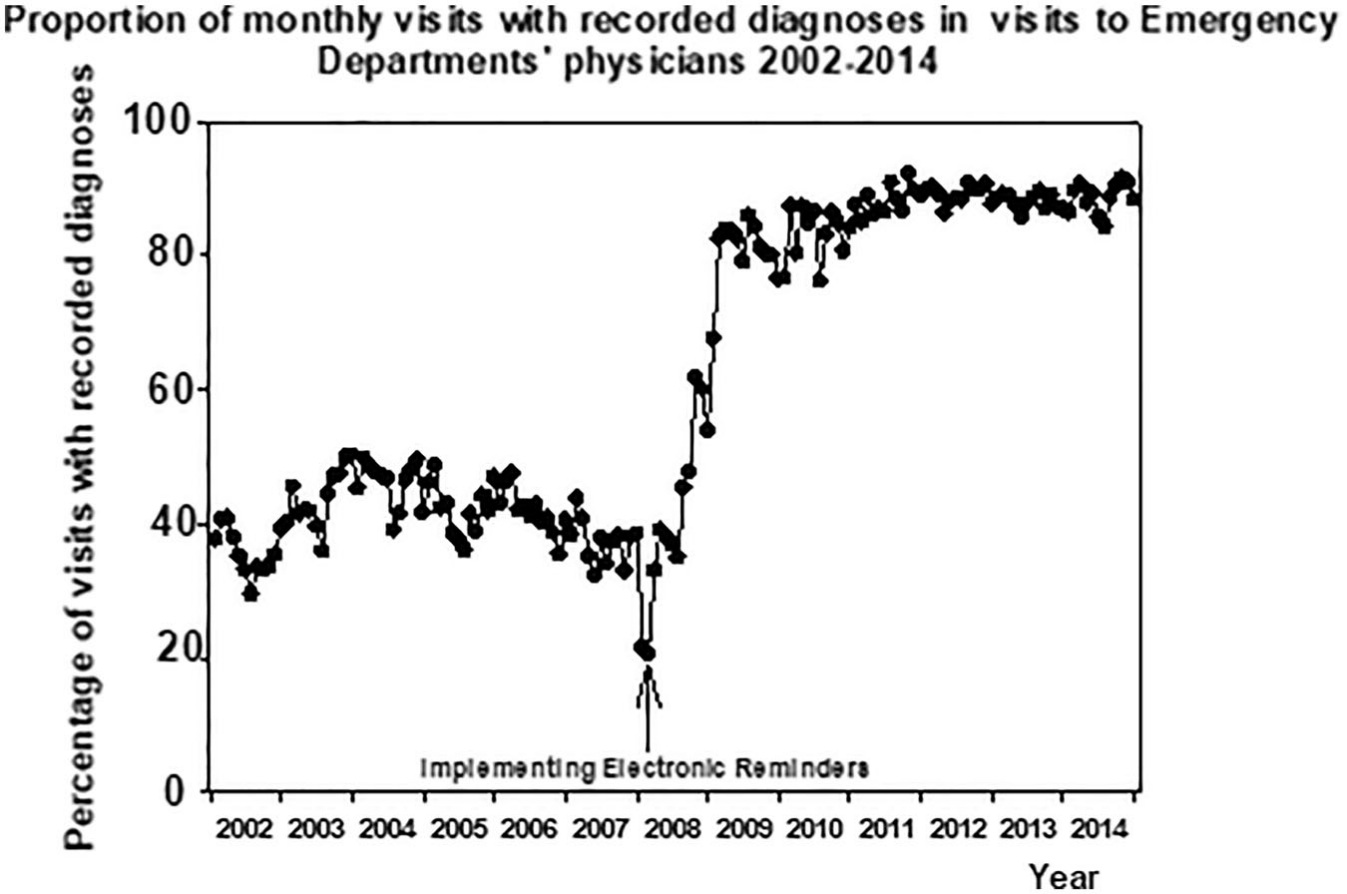
Monthly proportion of visits with recorded diagnoses to the physicians of the primary health care Emergency Department 2002–2014 in the city of Vantaa, Finland.

**Figure 3.**
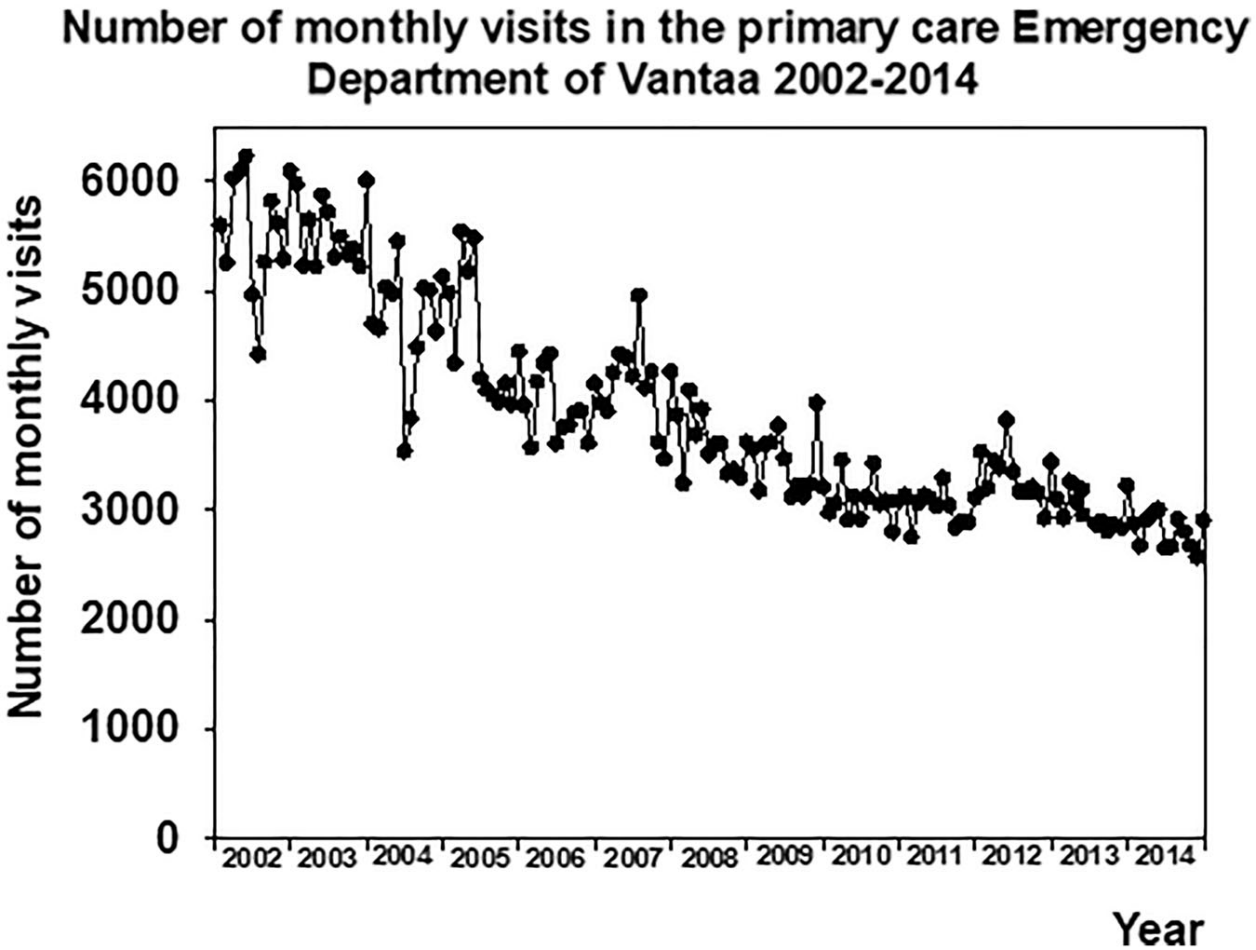
Number of monthly visits to the primary care emergency department 2002–2014.

There was no change in the monthly rate of recorded diagnoses before the installation of electronic reminders (−0.0097 ± 0.029%/month, *p* > 0.05). This rate of change increased to 3.56 ± 0.39%/month (*p* < 0.001) during the first year after the implementation of electronic reminders (Figure 2). During the next 6 years of the follow-up of the post-intervention period this increase continued (0.12 ± 0.016%/month, *p* < 0.001). The rate of change in the recording of diagnoses was at its highest during the first year after the intervention (*p* < 0.001 vs. before intervention or six last years of the follow-up). This rate of change was still higher during the six post-intervention years when compared with the pre-intervention period (*p* < 0.001, Figure 2). The number of monthly visits to the ED decreased during the follow-up (Figure 3).

### Association of electronic reminders with changes in the distribution of different diagnoses

Mental and behavioural disorders (group F), and injuries (groups S–Y) were more frequently recorded after installation of electronic reminders (Table 2). This was both the case with symptomatic diagnoses (group R) as with most of the other main diagnosis groups, too. Instead, proportions of respiratory diseases (group J), miscellaneous infections (groups A and B) and diseases of the eye and the adnexa, and the ear and mastoid process (group H) decreased after this intervention.

**Table 2.** The distribution of the main groups of 10th edition International Classification of Diseases (ICD-10) diagnoses before (2002–2007) and after (2009–2014) application of electronic reminders in the primary health care emergency department in the city of Vantaa, Finland.

ICD-10	Contents of diagnosis group	% of all diagnoses		% of all visits	
		Before electronic reminder (%)	After electronic reminder	Before electronic reminder (%)	After electronic reminder
A	Intestinal infectious diseases, bacterial infections, and viral infections of central nervous system	4.2	3.68%*	1.76	3.04%***
B	Other infections	0.93	0.68%*	0.39	0.56%**
C	Malignant neoplasms	0.11	0.11%	0.04	0.09%
D	Other neoplasms and carcinoma *in situ*	0.11	0.21%	0.04	0.17%***
E	Endocrine nutritional and metabolic diseases	0.34	0.78%***	0.14	0.65%***
F	Mental and behavioural disorders	3.49	6.31%***	1.46	5.21%***
G	Diseases of the nervous systems	1.56	1.91%*	0.65	1.58%***
H	Diseases of the eye and the adnexa, and the ear and mastoid process	11.64	6.74%***	4.86	5.57%***
I	Diseases of the circulatory system	2.21	3.95%***	0.92	3.2%***
J	Diseases of the respiratory system	25.36	12.64%***	10.59	10.44%
K	Diseases of the digestive system	2.47	3.14%***	1.03	2.6%***
L	Diseases of the skin and subcutaneous tissue	2.13	1.69%**	0.89	1.4%***
M	Diseases of the musculoskeletal system and connective tissue	8.80	7.62%***	3.67	6.3%***
N	Diseases of genitourinary system	3.47	4.72%***	1.45	3.9%***
O	Pregnancy, childbirth, and puerperium	0.34	0.39%	0.14	0.32%***
P	Certain conditions originating in the perinatal period	0.01	0.02%	0.00	0.02%
Q	Congenital malformations, deformations, and chromosomal abnormalities	0.03	0.02%	0.01	0.02%
R	Symptoms, signs and abnormal clinical and laboratory findings, not elsewhere classified	11.3	19.29%***	4.71	15.94%***
S	Injury, poisoning and certain other consequences of external causes, single body region	18.00	20.76%***	7.51	17.15%***
T	Injuries to multiple or unspecified body regions as well as poisoning and certain other consequences of external causes.	2.22	2.70%*	0.92	2.23%***
V	Transport accidents	0.02	0.16%**	0.01	0.12%***
W	Other external causes of accidental injury	0.21	0.65%***	0.09	0.53%***
X	Exposure to burning substances and related threads, venomous animals and plants, noxious substances, and forces of nature. Intentional self-harm and assault	0.14	0.39%***	0.06	0.32%***
Y	Events of undetermined intent, legal interventions, and operations of war, complications of medical care, sequelae of external causes of morbidity and mortality	0.1	0.21%*	0.04	0.17%***
Z	Factors influencing health status and contact with health services	0.80	1.2%***	0.33	0.99%***

*Stands for *p* < 0.05, **p < 0.01, and ***p < 0.001 before versus after, Χ^2^ test.

Both absolute numbers and relative proportions of all recorded ICD-10 code group R-diagnoses, such as abdominal and pelvic pain (837 ≥ 1850, 3.6% ≥ 5.8%), pain in throat and chest (290 ≥ 748, 1.3% ≥ 2.3%), malaise and fatigue (109 ≥ 549, 0.5% ≥ 1.7%), and abnormalities of breathing (182 ≥ 413, 0.8% ≥ 1.3%), increased after the installation of electronic reminders (Table 3). Diagnosis related to alcohol abuse (ICD-10 code F10) also increased (209 ≥ 885, 1.0% ≥ 2.8%) (Table 3). Various infections of upper respiratory airways were recorded less frequently after the installation of ERs (Table 3).

**Table 3.** Percentages and absolute numbers of the 20 most common 10th edition International Classification of Diseases (ICD-10) diagnosis groups 6 years before and after the implementation of electronic reminders during the follow-up 2002–2014.

ICD-10 code	Name of diagnosis group	%/year, before electronic reminders % of diagnoses	%/year, after electronic reminders % of diagnoses	%/year, before electronic reminders *% of all visits*	%/year after electronic reminders *% of all visits*	*N* before electronic reminders (N/year)	*N* after electronic reminders (N/year)
J06	Acute upper respiratory infections of multiple and unspecified sites	8.12 ± 2.20	3.90 ± 0.75***	3.35 ± 0.97	3.35 ± 0.52	1949 ± 780	1260 ± 290
R10	Abdominal and pelvic pain	3.64 ± 0.709	5.77 ± 0.76***	1.51 ± 0.37	5.00 ± 0.83***	837 ± 169	1850 ± 231***
H66	Suppurative and unspecified otitis media	6.30 ± 0.74	3.1 ± 0.5***	2.60 ± 0.381	2.63 ± 0.365	1484 ± 386	986 ± 181*
M54	Dorsalgia	4.70 ± 0.375	3.33 ± 0.44***	1.94 ± 0.293	2.86 ± 0.317***	1105 ± 260	1073 ± 177
S01	Open wound of head	2.34 ± 0.47	2.92 ± 0.21*	0.97 ± 0.23	2.53 ± 0.23***	535 ± 91	940 ± 86***
J20	Acute bronchitis	4.15 ± 0.99	1.55 ± 0.42***	1.72 ± 0.46	1.33 ± 0.31	994 ± 378	499 ± 140*
A09	Other gastroenteritis and colitis of infectious and unspecified origin	2.35 ± 0.38	1.68 ± 0.16**	0.97 ± 0.15	1.44 ± 0.1***	533(413–721)	543(490–578)
F10	Mental and behavioural disorder due to use of alcohol	1.00 ± 0.86	2.8 ± 0.4**	0.41 ± 0.32	2.38 ± 0.42***	209 ± 150	885 ± 146***
R07	Pain in throat and chest	1.29 ± 0.35	2.33 ± 0.25***	0.53 ± 0.13	2.02 ± 0.28***	290 ± 36	748 ± 75***
S93	Dislocation, sprain and strain of joints and ligaments at ankle and foot level	2.07 ± 0.14	1.51 ± 0.12***	0.85 ± 0.66	1.31 ± 0.14***	493(402–552)	482(453–525)
S61	Open wound of wrist and hand	1.76 ± 0.30	1.64 ± 0.16	0.73 ± 0.14	1.41 ± 0.17***	403 ± 43	528 ± 76
N30	Cystitis	1.56 ± 0.12	1.79 ± 00.17*	0.65 (0.62–0.66)	1.58 (1.36–1.68)**	360 ± 40	573 ± 27***
J03	Acute tonsillitis	2.51 (2.36–3.27)	0.87 (0.51–1.07)***	1.14 ± 0.22	0.69 ± 0.23**	657 ± 215	262 ± 94**
H10	Conjunctivitis	2.18 ± 0.36	1.05 ± 0.45***	0.91 (0.79–0.97)	0.86 (0.57–1.23)	508 ± 114	337 ± 141*
J01	Acute sinusitis	2.59 ± 1.02	0.68 ± 0.41*	1.06 ± 0.413	0.58 ± 0.313*	615 (304–958)	178 (143–286)**
M79	Other soft tissue disorders, not elsewhere classified	0.63 (0.48–0.69)	1.86 (1.34–2.20)**	0.24 (0.21–0.29)	1.65 (1.09–1.94)**	133 (114–161)	619 (437–691)**
S06	Intracranial injury	1.23 ± 0.12	1.21 ± 0.20	0.51 ± 0.07	1.05 ± 0.19***	283 ± 36	391 ± 82*
R53	Malaise and fatigue	0.49 ± 0.24	1.72 ± 0.39***	0.20 ± 0.10	1.49 ± 0.38***	109 ± 46	549 ± 110***
R06	Abnormalities of breathing	0.80 (0.54–1.12)	1.3 (1.19–1.41)**	0.33 ± 0.15	1.12 ± 0.16***	182 ± 79	413 ± 49***
S52	Fracture of forearm	0.68 (0.61–0.74)	1.22 (0.86–1.96)**	0.33 ± 0.15	1.12 ± 0.16***	161 ± 140	422 ± 272**

The data are expressed as mean ± SD or median (25–75% quartile range).

*Stands for *p* < 0.05, **p < 0.01, and ***p < 0.001 before versus after, *t* test or Mann–Whiney *U* test when appropriate.

## Discussion

Most of the recorded diagnoses in the ED were infections in the superior part of the respiratory system. Electronic reminders were effective in facilitating the recording of diagnoses. Especially the recording of symptomatic diagnoses (ICD-10 code group R-diagnoses), mental and behavioural disorders (group F), and injuries (groups S-Y) were enhanced after implementation of electronic reminders. Additionally, diagnoses related to alcohol abuse increased.

The strength of this study is that the present result reflects real clinical activity in primary health care EDs. Thus, these results are only applicable with certainty to primary health care EDs. Due to the retrospective setting, the participants were unaware of being studied. Lack of data concerning individual physicians and their behavior inhibits us from drawing conclusions about whether there were physicians who did not respond to this intervention or whether there were physicians who regularly recorded inappropriate diagnoses despite the electronic reminders. We cannot totally exclude secular trends contributing partly to the changes in diagnosis recording rates. In the time of the follow-up there were also other changes in the ED, such as application of ABCDE-triage from February 1st, 2004 [11] and its revised version [16] from February 1st 2008 [17], and a decrease of evening practices in the primary care of the Western part of Vantaa starting on June 1st 2005 [12]. Neither we know surely whether electronic reminder system was solely responsible for change in practice or how much increased recording was due to education. However, the change in recording diagnoses was abrupt and happened right after the electronic reminder was introduced. Thus, this has much larger impact than reminders guiding testing or prescribing [1]. Therefore, it is fair to conclude that the reminder played large role considering the fact that the diagnosis recording rate remained elevated throughout the remainder of the follow-up period. This is not to be interpreted that the individual feedback had no effect in terms of facilitating the change. However, there was considerable variation in the amount and frequency of feedback given to the doctors in the primary care of Vantaa [9,10] whereas the reminder was introduced systematically and simultaneously to all users in 2008.

In this context, it is important to notice that despite the rate of recorded diagnoses increasing with electronic reminders, categorizing patients with diagnoses *per se* does not automatically lead to better quality in the contents of recording because it does not guarantee that recorded diagnoses are clinically correct [18]. Thus, diagnosing itself does not directly lead to ‘better treatment’ or necessarily improve the quality of care experienced by the patients [19], although it may enhance the quality of treatment from the health care system’s point of view [1].

Eliciting the missing diagnosis recoding data with electronic reminders also altered the distribution of documented diagnoses in the primary health care ED. It appears that ED physicians increased the recording of group R diagnoses of ICD-10 -system. These codes refer to diagnoses which describe only the symptoms, signs and abnormal clinical findings while not suggesting any specific disease underlying them [20]. Thus, the physicians in the ED may not have reached a conclusion in terms of a specific diagnosis in all situations. As reported before [21,22], this fairly common with unscreened patients and therefore diagnosis recordings may have been neglected to some extent before the present intervention. Upon eliciting the missing diagnosis documentation with electronic reminders, physicians were more inclined to adapt to recording symptoms using the ICD-10 -system.

Analogously, recording the diagnosis for alcohol abuse in EDs is challenging for various reasons: the acceptance of alcohol in the culture of the Western world, and apathy or lack of skills on the part of the ED-staff, and denial on the part of the patient may decrease the recording of ICD-10 code F10 diagnoses in EDs [23,24]. Furthermore, alcohol-related diagnoses are easily stigmatizing [25]. Visiting an ED under the influence of alcohol has been a frequent reason for one-fifth of the hospital admissions into the wards of the secondary care department of the ED currently being studied [26]. Yet recording alcohol misuse as a reason to visit the ED was not common in the beginning of the follow-up. Thus, implementation of electronic reminders may have resulted in physicians gaining the confidence to record alcohol-related reasons for ED visits and the consequent increase in the use of that diagnosis. The same phenomenon may have explained the observed improvement in recording of mental and behavioural disorders (group F) which may also stigmatize patients easily [27].

Yet there may also have been secular trends affecting the observed change in the distribution of diagnosis recordings. Naturally, some diagnostic drift and changes in the population's health is expected over a 12-year period. Furthermore, the decrease in relative proportions, as well as absolute numbers, of diagnosis recordings of mild respiratory infections suggests that there may have been changes in the inclusion criteria of ED patients. Indeed, a change in the triage system, namely, the adoption of the so-called ‘reverse triage’, was initiated in the beginning of 2008 [17]. In this type of triage-method an ED tries to redirect patients with mild health disorders to office-hour general practitioners [17], thereby reducing the number of patients entering the primary health care ED [28]. Indirectly, this change in ICD-10 code J-group diagnoses recordings may suggest that by using ‘reverse triage’ the ED succeeded in reducing the amount of certain types of patients entering the facility, such as those with mild respiratory infections.

Although this study was performed in a primary health care ED, these results are in line with former studies suggesting the usefulness of electronic reminders in altering clinical practice in all EDs [2–7]. Especially with the present, relatively simple type of intervention targeted at improving the quality of clinical recording, the application of electronic reminders seemed to function well. Reminders have been suggested as being an effective tool when pursuing improvement of the quality of patient records [8]. The importance of this is further emphasized because the recording of diagnoses may ensure sufficient treatment actions, enhance planning activities and direct management of resources [29]. Improving the extent of diagnosis recordings of chronic diseases may improve the quality of care [29] including by improving adherence to guidelines [5]. Recording diagnoses promotes diagnostic thinking [30]. It may lead to better treatment outcomes and increased patient safety by enhancing rational judgement of treatment options [30]. Recording diagnoses is, to some extent, a prerequisite for the use of computer-based clinical decision support systems [30]. Educational functions are also supported by frequent recording of diagnoses [31].

There was considerable variation in the percentages of visits with specific recorded diagnoses, depending on whether the percentage was calculated using only the number of visits having recorded diagnoses, or all visits to the ED physicians as a denominator. There is an explanation for these discrepancies. Due to the novel triage methods applied [11,17] and centralization procedures in the ED [12], the number of visits in the ED decreased during the follow-up period [27]. This may have modulated considerably the proportions calculated from all visits, but not those calculated from visits with recorded diagnoses. Therefore, studying diagnosis recordings as a measure of function should always be interpreted cautiously and several variables should be examined instead of observing only one.

Electronic reminders may alter clinical practice in EDs. At least the quality in terms of the extent of recorded diagnoses data can be improved by using them. Electronic reminders were effective in enhancing the recording of symptomatic diagnoses. They were also found to be effective with regard to diagnoses that tended to be neglected before their implementation, such as diagnoses related to alcohol abuse. By enhancing the recording of diagnoses ERs may provide a tool to ensure treatment actions, planning activities and management of resources in EDs.

## Ethical permissions

The register holder (the health authorities of Vantaa) and the scientific ethical board of Vantaa City (TUTKE) granted permission (VD/8059/13.00.00/2016) to carry out the study.

## Supplementary Material

Supplemental Material
