# Supplementary material for: The influence of electronic reminders on recording diagnoses in a primary health care emergency department: a register-based study in a Finnish town
Source: Scand J Prim Health Care. 2021 Apr 14;39(2):113–22. doi: 10.1080/02813432.2021.1910449 (PMC8293956; doi:10.1080/02813432.2021.1910449)
Supplement: Supplemental Material [file IPRI_A_1910449_SM4837.docx]

**Table I** Cumulative percentage of visits to the primary health care emergency department physicians as a function of different recorded 10^th^ edition International Classification of Diseases (ICD-10)-diagnoses in the city of Vantaa, Finland. All diagnoses (full data) are shown.

| **Order** | **ICD-10 code** | **Number** | **Percentage** | **Cumulative** | **Order** | **ICD-10 code** | **Number** | **Percentage** | **Cumulative** | **Order** | **ICD-10 code** | **Number** | **Percentage** | **Cumulative** |
| --- | --- | --- | --- | --- | --- | --- | --- | --- | --- | --- | --- | --- | --- | --- |
| **1** | J06 | 20381 | 5,821 % | 5,821 % | **438** | S19 | 33 | 0,009 % | 98,211 % | **875** | L81 | 4 | 0,001 % | 99,780 % |
| **2** | R10 | 16843 | 4,810 % | 10,631 % | **439** | S11 | 33 | 0,009 % | 98,220 % | **876** | M21 | 4 | 0,001 % | 99,781 % |
| **3** | H66 | 15717 | 4,489 % | 15,120 % | **440** | R99 | 33 | 0,009 % | 98,230 % | **877** | N85 | 4 | 0,001 % | 99,782 % |
| **4** | M54 | 13845 | 3,954 % | 19,074 % | **441** | N83 | 33 | 0,009 % | 98,239 % | **878** | F94 | 4 | 0,001 % | 99,783 % |
| **5** | S01 | 9401 | 2,685 % | 21,759 % | **442** | I44 | 33 | 0,009 % | 98,249 % | **879** | H25 | 4 | 0,001 % | 99,784 % |
| **6** | J20 | 9306 | 2,658 % | 24,417 % | **443** | B33 | 33 | 0,009 % | 98,258 % | **880** | G61 | 4 | 0,001 % | 99,785 % |
| **7** | A09 | 6917 | 1,976 % | 26,393 % | **444** | N80 | 32 | 0,009 % | 98,267 % | **881** | I97 | 4 | 0,001 % | 99,786 % |
| **8** | F10 | 6879 | 1,965 % | 28,357 % | **445** | K14 | 32 | 0,009 % | 98,276 % | **882** | H55 | 4 | 0,001 % | 99,788 % |
| **9** | R07 | 6520 | 1,862 % | 30,220 % | **446** | F93 | 32 | 0,009 % | 98,286 % | **883** | I99 | 4 | 0,001 % | 99,789 % |
| **10** | S93 | 6160 | 1,759 % | 31,979 % | **447** | W00 | 31 | 0,009 % | 98,294 % | **884** | I72 | 4 | 0,001 % | 99,790 % |
| **11** | S61 | 5927 | 1,693 % | 33,672 % | **448** | R47 | 31 | 0,009 % | 98,303 % | **885** | F68 | 4 | 0,001 % | 99,791 % |
| **12** | N30 | 5919 | 1,690 % | 35,362 % | **449** | J41 | 31 | 0,009 % | 98,312 % | **886** | G90 | 4 | 0,001 % | 99,792 % |
| **13** | J03 | 5785 | 1,652 % | 37,014 % | **450** | G31 | 31 | 0,009 % | 98,321 % | **887** | H74 | 4 | 0,001 % | 99,793 % |
| **14** | H10 | 5499 | 1,571 % | 38,585 % | **451** | H68 | 31 | 0,009 % | 98,330 % | **888** | C92 | 4 | 0,001 % | 99,794 % |
| **15** | J01 | 5375 | 1,535 % | 40,120 % | **452** | I45 | 31 | 0,009 % | 98,339 % | **889** | D10 | 4 | 0,001 % | 99,796 % |
| **16** | M79 | 4508 | 1,288 % | 41,408 % | **453** | B80 | 31 | 0,009 % | 98,347 % | **890** | A28 | 4 | 0,001 % | 99,797 % |
| **17** | S06 | 4240 | 1,211 % | 42,619 % | **454** | K25 | 30 | 0,009 % | 98,356 % | **891** | F38 | 4 | 0,001 % | 99,798 % |
| **18** | R53 | 4078 | 1,165 % | 43,783 % | **455** | F50 | 30 | 0,009 % | 98,365 % | **892** | C09 | 4 | 0,001 % | 99,799 % |
| **19** | R06 | 3694 | 1,055 % | 44,838 % | **456** | I69 | 30 | 0,009 % | 98,373 % | **893** | C95 | 4 | 0,001 % | 99,800 % |
| **20** | S52 | 3672 | 1,049 % | 45,887 % | **457** | T54 | 29 | 0,008 % | 98,381 % | **894** | A92 | 4 | 0,001 % | 99,801 % |
| **21** | H65 | 3605 | 1,030 % | 46,917 % | **458** | X23 | 29 | 0,008 % | 98,390 % | **895** | B06 | 4 | 0,001 % | 99,802 % |
| **22** | R42 | 3516 | 1,004 % | 47,921 % | **459** | Y96 | 29 | 0,008 % | 98,398 % | **896** | C39 | 4 | 0,001 % | 99,804 % |
| **23** | N39 | 3475 | 0,992 % | 48,913 % | **460** | ZA7 | 29 | 0,008 % | 98,406 % | **897** | E73 | 4 | 0,001 % | 99,805 % |
| **24** | J18 | 3361 | 0,960 % | 49,873 % | **461** | R19 | 29 | 0,008 % | 98,415 % | **898** | D53 | 4 | 0,001 % | 99,806 % |
| **25** | R51 | 3221 | 0,920 % | 50,793 % | **462** | N95 | 29 | 0,008 % | 98,423 % | **899** | A06 | 4 | 0,001 % | 99,807 % |
| **26** | F32 | 3062 | 0,875 % | 51,668 % | **463** | N43 | 29 | 0,008 % | 98,431 % | **900** | W26 | 3 | 0,001 % | 99,808 % |
| **27** | S62 | 3055 | 0,873 % | 52,540 % | **464** | N18 | 29 | 0,008 % | 98,439 % | **901** | Y83 | 3 | 0,001 % | 99,809 % |
| **28** | T74 | 2888 | 0,825 % | 53,365 % | **465** | K36 | 29 | 0,008 % | 98,448 % | **902** | W40 | 3 | 0,001 % | 99,810 % |
| **29** | S60 | 2768 | 0,791 % | 54,156 % | **466** | L89 | 29 | 0,008 % | 98,456 % | **903** | Y11 | 3 | 0,001 % | 99,810 % |
| **30** | J04 | 2760 | 0,788 % | 54,944 % | **467** | H33 | 29 | 0,008 % | 98,464 % | **904** | X41 | 3 | 0,001 % | 99,811 % |
| **31** | A46 | 2669 | 0,762 % | 55,706 % | **468** | J12 | 29 | 0,008 % | 98,473 % | **905** | Y13 | 3 | 0,001 % | 99,812 % |
| **32** | J02 | 2630 | 0,751 % | 56,457 % | **469** | H18 | 29 | 0,008 % | 98,481 % | **906** | T95 | 3 | 0,001 % | 99,813 % |
| **33** | R50 | 2615 | 0,747 % | 57,204 % | **470** | A98 | 29 | 0,008 % | 98,489 % | **907** | X95 | 3 | 0,001 % | 99,814 % |
| **34** | F41 | 2605 | 0,744 % | 57,948 % | **471** | A87 | 29 | 0,008 % | 98,497 % | **908** | Z89 | 3 | 0,001 % | 99,815 % |
| **35** | S63 | 2292 | 0,655 % | 58,603 % | **472** | T58 | 28 | 0,008 % | 98,505 % | **909** | X91 | 3 | 0,001 % | 99,815 % |
| **36** | S82 | 2193 | 0,626 % | 59,229 % | **473** | L70 | 28 | 0,008 % | 98,513 % | **910** | Y10 | 3 | 0,001 % | 99,816 % |
| **37** | J45 | 2024 | 0,578 % | 59,807 % | **474** | M80 | 28 | 0,008 % | 98,521 % | **911** | Z10 | 3 | 0,001 % | 99,817 % |
| **38** | S42 | 2009 | 0,574 % | 60,381 % | **475** | J69 | 28 | 0,008 % | 98,529 % | **912** | V80 | 3 | 0,001 % | 99,818 % |
| **39** | G43 | 1919 | 0,548 % | 60,929 % | **476** | H91 | 28 | 0,008 % | 98,537 % | **913** | W02 | 3 | 0,001 % | 99,819 % |
| **40** | S80 | 1876 | 0,536 % | 61,465 % | **477** | X49 | 27 | 0,008 % | 98,545 % | **914** | X46 | 3 | 0,001 % | 99,820 % |
| **41** | R11 | 1816 | 0,519 % | 61,983 % | **478** | X78 | 27 | 0,008 % | 98,553 % | **915** | Y15 | 3 | 0,001 % | 99,821 % |
| **42** | T78 | 1795 | 0,513 % | 62,496 % | **479** | G25 | 27 | 0,008 % | 98,561 % | **916** | Z65 | 3 | 0,001 % | 99,821 % |
| **43** | S90 | 1770 | 0,506 % | 63,002 % | **480** | Z93 | 26 | 0,007 % | 98,568 % | **917** | Z34 | 3 | 0,001 % | 99,822 % |
| **44** | S92 | 1740 | 0,497 % | 63,499 % | **481** | W10 | 26 | 0,007 % | 98,575 % | **918** | X85 | 3 | 0,001 % | 99,823 % |
| **45** | I49 | 1676 | 0,479 % | 63,977 % | **482** | T80 | 26 | 0,007 % | 98,583 % | **919** | Y16 | 3 | 0,001 % | 99,824 % |
| **46** | I10 | 1668 | 0,476 % | 64,454 % | **483** | T33 | 26 | 0,007 % | 98,590 % | **920** | W87 | 3 | 0,001 % | 99,825 % |
| **47** | S00 | 1594 | 0,455 % | 64,909 % | **484** | S68 | 26 | 0,007 % | 98,598 % | **921** | Y42 | 3 | 0,001 % | 99,826 % |
| **48** | L02 | 1586 | 0,453 % | 65,362 % | **485** | O22 | 26 | 0,007 % | 98,605 % | **922** | Y07 | 3 | 0,001 % | 99,827 % |
| **49** | J11 | 1583 | 0,452 % | 65,814 % | **486** | M47 | 26 | 0,007 % | 98,613 % | **923** | Z47 | 3 | 0,001 % | 99,827 % |
| **50** | R05 | 1559 | 0,445 % | 66,259 % | **487** | J38 | 26 | 0,007 % | 98,620 % | **924** | X90 | 3 | 0,001 % | 99,828 % |
| **51** | R55 | 1549 | 0,442 % | 66,702 % | **488** | K07 | 26 | 0,007 % | 98,627 % | **925** | W05 | 3 | 0,001 % | 99,829 % |
| **52** | R52 | 1543 | 0,441 % | 67,142 % | **489** | W89 | 25 | 0,007 % | 98,635 % | **926** | Y88 | 3 | 0,001 % | 99,830 % |
| **53** | H60 | 1532 | 0,438 % | 67,580 % | **490** | T30 | 25 | 0,007 % | 98,642 % | **927** | W07 | 3 | 0,001 % | 99,831 % |
| **54** | R04 | 1526 | 0,436 % | 68,016 % | **491** | R63 | 25 | 0,007 % | 98,649 % | **928** | V02 | 3 | 0,001 % | 99,832 % |
| **55** | R56 | 1500 | 0,428 % | 68,444 % | **492** | L21 | 25 | 0,007 % | 98,656 % | **929** | Q85 | 3 | 0,001 % | 99,833 % |
| **56** | L50 | 1497 | 0,428 % | 68,872 % | **493** | K83 | 25 | 0,007 % | 98,663 % | **930** | R96 | 3 | 0,001 % | 99,833 % |
| **57** | M75 | 1462 | 0,418 % | 69,289 % | **494** | K02 | 25 | 0,007 % | 98,670 % | **931** | R80 | 3 | 0,001 % | 99,834 % |
| **58** | N10 | 1457 | 0,416 % | 69,705 % | **495** | I73 | 25 | 0,007 % | 98,677 % | **932** | P54 | 3 | 0,001 % | 99,835 % |
| **59** | S83 | 1436 | 0,410 % | 70,115 % | **496** | T59 | 24 | 0,007 % | 98,684 % | **933** | Q21 | 3 | 0,001 % | 99,836 % |
| **60** | S43 | 1430 | 0,408 % | 70,524 % | **497** | R78 | 24 | 0,007 % | 98,691 % | **934** | Q78 | 3 | 0,001 % | 99,837 % |
| **61** | A08 | 1401 | 0,400 % | 70,924 % | **498** | F84 | 24 | 0,007 % | 98,698 % | **935** | P78 | 3 | 0,001 % | 99,838 % |
| **62** | L03 | 1398 | 0,399 % | 71,323 % | **499** | X64 | 23 | 0,007 % | 98,704 % | **936** | S04 | 3 | 0,001 % | 99,839 % |
| **63** | M53 | 1393 | 0,398 % | 71,721 % | **500** | ZA8 | 23 | 0,007 % | 98,711 % | **937** | Q43 | 3 | 0,001 % | 99,839 % |
| **64** | S20 | 1371 | 0,392 % | 72,113 % | **501** | W86 | 23 | 0,007 % | 98,718 % | **938** | Q77 | 3 | 0,001 % | 99,840 % |
| **65** | G44 | 1358 | 0,388 % | 72,501 % | **502** | T19 | 23 | 0,007 % | 98,724 % | **939** | K90 | 3 | 0,001 % | 99,841 % |
| **66** | I48 | 1260 | 0,360 % | 72,860 % | **503** | J96 | 23 | 0,007 % | 98,731 % | **940** | K31 | 3 | 0,001 % | 99,842 % |
| **67** | S81 | 1206 | 0,344 % | 73,205 % | **504** | I51 | 23 | 0,007 % | 98,737 % | **941** | N00 | 3 | 0,001 % | 99,843 % |
| **68** | Z72 | 1160 | 0,331 % | 73,536 % | **505** | C18 | 23 | 0,007 % | 98,744 % | **942** | L63 | 3 | 0,001 % | 99,844 % |
| **69** | K59 | 1147 | 0,328 % | 73,864 % | **506** | T01 | 22 | 0,006 % | 98,750 % | **943** | L45 | 3 | 0,001 % | 99,845 % |
| **70** | H81 | 1135 | 0,324 % | 74,188 % | **507** | K72 | 22 | 0,006 % | 98,756 % | **944** | L91 | 3 | 0,001 % | 99,845 % |
| **71** | S22 | 1085 | 0,310 % | 74,498 % | **508** | L44 | 22 | 0,006 % | 98,763 % | **945** | O46 | 3 | 0,001 % | 99,846 % |
| **72** | T15 | 1066 | 0,304 % | 74,802 % | **509** | N50 | 22 | 0,006 % | 98,769 % | **946** | L90 | 3 | 0,001 % | 99,847 % |
| **73** | I50 | 996 | 0,284 % | 75,087 % | **510** | O26 | 22 | 0,006 % | 98,775 % | **947** | M81 | 3 | 0,001 % | 99,848 % |
| **74** | I20 | 992 | 0,283 % | 75,370 % | **511** | G62 | 22 | 0,006 % | 98,782 % | **948** | L75 | 3 | 0,001 % | 99,849 % |
| **75** | G40 | 980 | 0,280 % | 75,650 % | **512** | I42 | 22 | 0,006 % | 98,788 % | **949** | N91 | 3 | 0,001 % | 99,850 % |
| **76** | F20 | 939 | 0,268 % | 75,918 % | **513** | G41 | 22 | 0,006 % | 98,794 % | **950** | M30 | 3 | 0,001 % | 99,851 % |
| **77** | S91 | 936 | 0,267 % | 76,185 % | **514** | A56 | 22 | 0,006 % | 98,800 % | **951** | K09 | 3 | 0,001 % | 99,851 % |
| **78** | K30 | 923 | 0,264 % | 76,449 % | **515** | N64 | 21 | 0,006 % | 98,806 % | **952** | H45 | 3 | 0,001 % | 99,852 % |
| **79** | R00 | 914 | 0,261 % | 76,710 % | **516** | N49 | 21 | 0,006 % | 98,812 % | **953** | F83 | 3 | 0,001 % | 99,853 % |
| **80** | F43 | 913 | 0,261 % | 76,971 % | **517** | J35 | 21 | 0,006 % | 98,818 % | **954** | J17 | 3 | 0,001 % | 99,854 % |
| **81** | N20 | 893 | 0,255 % | 77,226 % | **518** | F16 | 21 | 0,006 % | 98,824 % | **955** | I46 | 3 | 0,001 % | 99,855 % |
| **82** | M23 | 889 | 0,254 % | 77,480 % | **519** | C25 | 21 | 0,006 % | 98,830 % | **956** | G04 | 3 | 0,001 % | 99,856 % |
| **83** | S50 | 851 | 0,243 % | 77,723 % | **520** | F44 | 21 | 0,006 % | 98,836 % | **957** | F79 | 3 | 0,001 % | 99,857 % |
| **84** | I80 | 843 | 0,241 % | 77,964 % | **521** | T31 | 20 | 0,006 % | 98,842 % | **958** | G64 | 3 | 0,001 % | 99,857 % |
| **85** | S51 | 813 | 0,232 % | 78,196 % | **522** | M20 | 20 | 0,006 % | 98,848 % | **959** | G70 | 3 | 0,001 % | 99,858 % |
| **86** | F05 | 811 | 0,232 % | 78,427 % | **523** | N81 | 20 | 0,006 % | 98,854 % | **960** | G55 | 3 | 0,001 % | 99,859 % |
| **87** | S30 | 799 | 0,228 % | 78,656 % | **524** | N11 | 20 | 0,006 % | 98,859 % | **961** | E28 | 3 | 0,001 % | 99,860 % |
| **88** | H92 | 790 | 0,226 % | 78,881 % | **525** | G58 | 20 | 0,006 % | 98,865 % | **962** | F17 | 3 | 0,001 % | 99,861 % |
| **89** | E87 | 767 | 0,219 % | 79,100 % | **526** | J81 | 20 | 0,006 % | 98,871 % | **963** | C02 | 3 | 0,001 % | 99,862 % |
| **90** | R60 | 751 | 0,214 % | 79,315 % | **527** | B36 | 20 | 0,006 % | 98,876 % | **964** | D43 | 3 | 0,001 % | 99,863 % |
| **91** | K57 | 742 | 0,212 % | 79,527 % | **528** | Z02 | 19 | 0,005 % | 98,882 % | **965** | A18 | 3 | 0,001 % | 99,863 % |
| **92** | J44 | 718 | 0,205 % | 79,732 % | **529** | R29 | 19 | 0,005 % | 98,887 % | **966** | D45 | 3 | 0,001 % | 99,864 % |
| **93** | H16 | 692 | 0,198 % | 79,929 % | **530** | N02 | 19 | 0,005 % | 98,893 % | **967** | E89 | 3 | 0,001 % | 99,865 % |
| **94** | S86 | 686 | 0,196 % | 80,125 % | **531** | K44 | 19 | 0,005 % | 98,898 % | **968** | D46 | 3 | 0,001 % | 99,866 % |
| **95** | S13 | 684 | 0,195 % | 80,321 % | **532** | L97 | 19 | 0,005 % | 98,904 % | **969** | D23 | 3 | 0,001 % | 99,867 % |
| **96** | K80 | 669 | 0,191 % | 80,512 % | **533** | H40 | 19 | 0,005 % | 98,909 % | **970** | A31 | 3 | 0,001 % | 99,868 % |
| **97** | M77 | 665 | 0,190 % | 80,702 % | **534** | E13 | 19 | 0,005 % | 98,914 % | **971** | D25 | 3 | 0,001 % | 99,869 % |
| **98** | I84 | 662 | 0,189 % | 80,891 % | **535** | D68 | 19 | 0,005 % | 98,920 % | **972** | A64 | 3 | 0,001 % | 99,869 % |
| **99** | J15 | 662 | 0,189 % | 81,080 % | **536** | T50 | 18 | 0,005 % | 98,925 % | **973** | C38 | 3 | 0,001 % | 99,870 % |
| **100** | S40 | 658 | 0,188 % | 81,268 % | **537** | Z20 | 18 | 0,005 % | 98,930 % | **974** | B77 | 3 | 0,001 % | 99,871 % |
| **101** | S70 | 654 | 0,187 % | 81,455 % | **538** | Z86 | 18 | 0,005 % | 98,935 % | **975** | D29 | 3 | 0,001 % | 99,872 % |
| **102** | K35 | 651 | 0,186 % | 81,640 % | **539** | R32 | 18 | 0,005 % | 98,940 % | **976** | Z92 | 2 | 0,001 % | 99,873 % |
| **103** | K29 | 647 | 0,185 % | 81,825 % | **540** | L71 | 18 | 0,005 % | 98,946 % | **977** | Y08 | 2 | 0,001 % | 99,873 % |
| **104** | S89 | 638 | 0,182 % | 82,007 % | **541** | G03 | 18 | 0,005 % | 98,951 % | **978** | Y09 | 2 | 0,001 % | 99,874 % |
| **105** | K92 | 627 | 0,179 % | 82,187 % | **542** | F90 | 18 | 0,005 % | 98,956 % | **979** | Z48 | 2 | 0,001 % | 99,874 % |
| **106** | F51 | 612 | 0,175 % | 82,361 % | **543** | K00 | 18 | 0,005 % | 98,961 % | **980** | T41 | 2 | 0,001 % | 99,875 % |
| **107** | B01 | 591 | 0,169 % | 82,530 % | **544** | H15 | 18 | 0,005 % | 98,966 % | **981** | X00 | 2 | 0,001 % | 99,875 % |
| **108** | G45 | 587 | 0,168 % | 82,698 % | **545** | E06 | 18 | 0,005 % | 98,971 % | **982** | Z29 | 2 | 0,001 % | 99,876 % |
| **109** | R25 | 580 | 0,166 % | 82,863 % | **546** | D62 | 18 | 0,005 % | 98,976 % | **983** | V11 | 2 | 0,001 % | 99,877 % |
| **110** | A49 | 578 | 0,165 % | 83,028 % | **547** | Z60 | 17 | 0,005 % | 98,981 % | **984** | X40 | 2 | 0,001 % | 99,877 % |
| **111** | R33 | 566 | 0,162 % | 83,190 % | **548** | T92 | 17 | 0,005 % | 98,986 % | **985** | Y47 | 2 | 0,001 % | 99,878 % |
| **112** | S05 | 563 | 0,161 % | 83,351 % | **549** | V19 | 17 | 0,005 % | 98,991 % | **986** | W42 | 2 | 0,001 % | 99,878 % |
| **113** | S99 | 552 | 0,158 % | 83,509 % | **550** | L00 | 17 | 0,005 % | 98,996 % | **987** | W12 | 2 | 0,001 % | 99,879 % |
| **114** | F31 | 545 | 0,156 % | 83,664 % | **551** | N99 | 17 | 0,005 % | 99,001 % | **988** | X59 | 2 | 0,001 % | 99,879 % |
| **115** | M25 | 541 | 0,155 % | 83,819 % | **552** | M35 | 17 | 0,005 % | 99,006 % | **989** | Y12 | 2 | 0,001 % | 99,880 % |
| **116** | M70 | 538 | 0,154 % | 83,972 % | **553** | G53 | 17 | 0,005 % | 99,010 % | **990** | Y84 | 2 | 0,001 % | 99,881 % |
| **117** | L01 | 535 | 0,153 % | 84,125 % | **554** | C90 | 17 | 0,005 % | 99,015 % | **991** | T86 | 2 | 0,001 % | 99,881 % |
| **118** | L30 | 527 | 0,151 % | 84,276 % | **555** | X09 | 16 | 0,005 % | 99,020 % | **992** | T60 | 2 | 0,001 % | 99,882 % |
| **119** | S72 | 498 | 0,142 % | 84,418 % | **556** | W09 | 16 | 0,005 % | 99,024 % | **993** | W85 | 2 | 0,001 % | 99,882 % |
| **120** | Z03 | 497 | 0,142 % | 84,560 % | **557** | P39 | 16 | 0,005 % | 99,029 % | **994** | X99 | 2 | 0,001 % | 99,883 % |
| **121** | R12 | 487 | 0,139 % | 84,699 % | **558** | L73 | 16 | 0,005 % | 99,034 % | **995** | T45 | 2 | 0,001 % | 99,883 % |
| **122** | M65 | 486 | 0,139 % | 84,838 % | **559** | L98 | 16 | 0,005 % | 99,038 % | **996** | Z45 | 2 | 0,001 % | 99,884 % |
| **123** | S46 | 484 | 0,138 % | 84,976 % | **560** | F80 | 16 | 0,005 % | 99,043 % | **997** | Y60 | 2 | 0,001 % | 99,885 % |
| **124** | L08 | 476 | 0,136 % | 85,112 % | **561** | K10 | 16 | 0,005 % | 99,047 % | **998** | V10 | 2 | 0,001 % | 99,885 % |
| **125** | Z00 | 473 | 0,135 % | 85,247 % | **562** | H03 | 16 | 0,005 % | 99,052 % | **999** | Y63 | 2 | 0,001 % | 99,886 % |
| **126** | B02 | 472 | 0,135 % | 85,382 % | **563** | G54 | 16 | 0,005 % | 99,056 % | **1000** | T57 | 2 | 0,001 % | 99,886 % |
| **127** | M10 | 467 | 0,133 % | 85,515 % | **564** | H70 | 16 | 0,005 % | 99,061 % | **1001** | Y85 | 2 | 0,001 % | 99,887 % |
| **128** | J30 | 450 | 0,129 % | 85,644 % | **565** | F28 | 16 | 0,005 % | 99,066 % | **1002** | Y45 | 2 | 0,001 % | 99,887 % |
| **129** | Z04 | 448 | 0,128 % | 85,772 % | **566** | F02 | 16 | 0,005 % | 99,070 % | **1003** | R90 | 2 | 0,001 % | 99,888 % |
| **130** | B34 | 443 | 0,127 % | 85,898 % | **567** | W44 | 15 | 0,004 % | 99,074 % | **1004** | Q61 | 2 | 0,001 % | 99,889 % |
| **131** | R31 | 442 | 0,126 % | 86,024 % | **568** | R46 | 15 | 0,004 % | 99,079 % | **1005** | O90 | 2 | 0,001 % | 99,889 % |
| **132** | M62 | 440 | 0,126 % | 86,150 % | **569** | S57 | 15 | 0,004 % | 99,083 % | **1006** | R27 | 2 | 0,001 % | 99,890 % |
| **133** | N48 | 436 | 0,125 % | 86,275 % | **570** | S65 | 15 | 0,004 % | 99,087 % | **1007** | Q65 | 2 | 0,001 % | 99,890 % |
| **134** | T23 | 430 | 0,123 % | 86,397 % | **571** | L13 | 15 | 0,004 % | 99,091 % | **1008** | T12 | 2 | 0,001 % | 99,891 % |
| **135** | S69 | 421 | 0,120 % | 86,518 % | **572** | K63 | 15 | 0,004 % | 99,096 % | **1009** | S38 | 2 | 0,001 % | 99,891 % |
| **136** | S53 | 411 | 0,117 % | 86,635 % | **573** | H62 | 15 | 0,004 % | 99,100 % | **1010** | P37 | 2 | 0,001 % | 99,892 % |
| **137** | R14 | 403 | 0,115 % | 86,750 % | **574** | I11 | 15 | 0,004 % | 99,104 % | **1011** | P57 | 2 | 0,001 % | 99,893 % |
| **138** | W55 | 400 | 0,114 % | 86,864 % | **575** | H82 | 15 | 0,004 % | 99,109 % | **1012** | T02 | 2 | 0,001 % | 99,893 % |
| **139** | I47 | 400 | 0,114 % | 86,979 % | **576** | H19 | 15 | 0,004 % | 99,113 % | **1013** | S85 | 2 | 0,001 % | 99,894 % |
| **140** | F29 | 400 | 0,114 % | 87,093 % | **577** | J98 | 15 | 0,004 % | 99,117 % | **1014** | S45 | 2 | 0,001 % | 99,894 % |
| **141** | K85 | 393 | 0,112 % | 87,205 % | **578** | J37 | 15 | 0,004 % | 99,121 % | **1015** | S87 | 2 | 0,001 % | 99,895 % |
| **142** | T14 | 387 | 0,111 % | 87,316 % | **579** | I24 | 15 | 0,004 % | 99,126 % | **1016** | S25 | 2 | 0,001 % | 99,895 % |
| **143** | X44 | 386 | 0,110 % | 87,426 % | **580** | X65 | 14 | 0,004 % | 99,130 % | **1017** | R02 | 2 | 0,001 % | 99,896 % |
| **144** | J22 | 386 | 0,110 % | 87,536 % | **581** | X58 | 14 | 0,004 % | 99,134 % | **1018** | Q55 | 2 | 0,001 % | 99,897 % |
| **145** | S02 | 384 | 0,110 % | 87,646 % | **582** | V89 | 14 | 0,004 % | 99,138 % | **1019** | Q40 | 2 | 0,001 % | 99,897 % |
| **146** | R41 | 380 | 0,109 % | 87,754 % | **583** | S16 | 14 | 0,004 % | 99,142 % | **1020** | Q72 | 2 | 0,001 % | 99,898 % |
| **147** | S66 | 376 | 0,107 % | 87,862 % | **584** | N22 | 14 | 0,004 % | 99,146 % | **1021** | Q87 | 2 | 0,001 % | 99,898 % |
| **148** | W19 | 359 | 0,103 % | 87,964 % | **585** | M89 | 14 | 0,004 % | 99,150 % | **1022** | Q18 | 2 | 0,001 % | 99,899 % |
| **149** | K21 | 359 | 0,103 % | 88,067 % | **586** | F63 | 14 | 0,004 % | 99,154 % | **1023** | T34 | 2 | 0,001 % | 99,899 % |
| **150** | M17 | 357 | 0,102 % | 88,169 % | **587** | D17 | 14 | 0,004 % | 99,158 % | **1024** | S55 | 2 | 0,001 % | 99,900 % |
| **151** | H00 | 355 | 0,101 % | 88,270 % | **588** | E05 | 14 | 0,004 % | 99,162 % | **1025** | S35 | 2 | 0,001 % | 99,901 % |
| **152** | W54 | 353 | 0,101 % | 88,371 % | **589** | A37 | 14 | 0,004 % | 99,166 % | **1026** | R87 | 2 | 0,001 % | 99,901 % |
| **153** | R44 | 345 | 0,099 % | 88,470 % | **590** | E03 | 14 | 0,004 % | 99,170 % | **1027** | N62 | 2 | 0,001 % | 99,902 % |
| **154** | S32 | 344 | 0,098 % | 88,568 % | **591** | B88 | 14 | 0,004 % | 99,174 % | **1028** | N04 | 2 | 0,001 % | 99,902 % |
| **155** | E16 | 344 | 0,098 % | 88,666 % | **592** | T70 | 13 | 0,004 % | 99,177 % | **1029** | O16 | 2 | 0,001 % | 99,903 % |
| **156** | I63 | 340 | 0,097 % | 88,763 % | **593** | P92 | 13 | 0,004 % | 99,181 % | **1030** | M91 | 2 | 0,001 % | 99,903 % |
| **157** | H11 | 340 | 0,097 % | 88,860 % | **594** | K76 | 13 | 0,004 % | 99,185 % | **1031** | O24 | 2 | 0,001 % | 99,904 % |
| **158** | W57 | 339 | 0,097 % | 88,957 % | **595** | L52 | 13 | 0,004 % | 99,189 % | **1032** | N82 | 2 | 0,001 % | 99,905 % |
| **159** | X69 | 326 | 0,093 % | 89,050 % | **596** | N32 | 13 | 0,004 % | 99,192 % | **1033** | N05 | 2 | 0,001 % | 99,905 % |
| **160** | H53 | 317 | 0,091 % | 89,141 % | **597** | F92 | 13 | 0,004 % | 99,196 % | **1034** | K55 | 2 | 0,001 % | 99,906 % |
| **161** | J36 | 315 | 0,090 % | 89,231 % | **598** | I67 | 13 | 0,004 % | 99,200 % | **1035** | M41 | 2 | 0,001 % | 99,906 % |
| **162** | R20 | 312 | 0,089 % | 89,320 % | **599** | G93 | 13 | 0,004 % | 99,203 % | **1036** | O07 | 2 | 0,001 % | 99,907 % |
| **163** | J21 | 302 | 0,086 % | 89,406 % | **600** | F06 | 13 | 0,004 % | 99,207 % | **1037** | K71 | 2 | 0,001 % | 99,907 % |
| **164** | K52 | 301 | 0,086 % | 89,492 % | **601** | B09 | 13 | 0,004 % | 99,211 % | **1038** | O08 | 2 | 0,001 % | 99,908 % |
| **165** | K40 | 299 | 0,085 % | 89,577 % | **602** | C71 | 13 | 0,004 % | 99,215 % | **1039** | O36 | 2 | 0,001 % | 99,909 % |
| **166** | J00 | 294 | 0,084 % | 89,661 % | **603** | Z75 | 12 | 0,003 % | 99,218 % | **1040** | L54 | 2 | 0,001 % | 99,909 % |
| **167** | J46 | 289 | 0,083 % | 89,744 % | **604** | Z98 | 12 | 0,003 % | 99,221 % | **1041** | O13 | 2 | 0,001 % | 99,910 % |
| **168** | H57 | 289 | 0,083 % | 89,826 % | **605** | T90 | 12 | 0,003 % | 99,225 % | **1042** | M33 | 2 | 0,001 % | 99,910 % |
| **169** | E11 | 287 | 0,082 % | 89,908 % | **606** | W22 | 12 | 0,003 % | 99,228 % | **1043** | I33 | 2 | 0,001 % | 99,911 % |
| **170** | K56 | 286 | 0,082 % | 89,990 % | **607** | Z54 | 12 | 0,003 % | 99,232 % | **1044** | F71 | 2 | 0,001 % | 99,911 % |
| **171** | V49 | 282 | 0,081 % | 90,071 % | **608** | S64 | 12 | 0,003 % | 99,235 % | **1045** | I38 | 2 | 0,001 % | 99,912 % |
| **172** | N76 | 279 | 0,080 % | 90,150 % | **609** | R01 | 12 | 0,003 % | 99,239 % | **1046** | H22 | 2 | 0,001 % | 99,913 % |
| **173** | S23 | 277 | 0,079 % | 90,229 % | **610** | M46 | 12 | 0,003 % | 99,242 % | **1047** | I31 | 2 | 0,001 % | 99,913 % |
| **174** | S71 | 270 | 0,077 % | 90,307 % | **611** | M84 | 12 | 0,003 % | 99,245 % | **1048** | H50 | 2 | 0,001 % | 99,914 % |
| **175** | R03 | 269 | 0,077 % | 90,383 % | **612** | G91 | 12 | 0,003 % | 99,249 % | **1049** | G98 | 2 | 0,001 % | 99,914 % |
| **176** | M13 | 266 | 0,076 % | 90,459 % | **613** | G95 | 12 | 0,003 % | 99,252 % | **1050** | H52 | 2 | 0,001 % | 99,915 % |
| **177** | J39 | 265 | 0,076 % | 90,535 % | **614** | I40 | 12 | 0,003 % | 99,256 % | **1051** | H36 | 2 | 0,001 % | 99,915 % |
| **178** | F23 | 265 | 0,076 % | 90,611 % | **615** | H02 | 12 | 0,003 % | 99,259 % | **1052** | J85 | 2 | 0,001 % | 99,916 % |
| **179** | L20 | 260 | 0,074 % | 90,685 % | **616** | F61 | 12 | 0,003 % | 99,263 % | **1053** | G21 | 2 | 0,001 % | 99,917 % |
| **180** | K11 | 259 | 0,074 % | 90,759 % | **617** | E66 | 12 | 0,003 % | 99,266 % | **1054** | H46 | 2 | 0,001 % | 99,917 % |
| **181** | O20 | 258 | 0,074 % | 90,833 % | **618** | A63 | 12 | 0,003 % | 99,269 % | **1055** | J61 | 2 | 0,001 % | 99,918 % |
| **182** | I21 | 257 | 0,073 % | 90,906 % | **619** | W50 | 11 | 0,003 % | 99,273 % | **1056** | C76 | 2 | 0,001 % | 99,918 % |
| **183** | S76 | 254 | 0,073 % | 90,979 % | **620** | V03 | 11 | 0,003 % | 99,276 % | **1057** | C32 | 2 | 0,001 % | 99,919 % |
| **184** | K62 | 252 | 0,072 % | 91,051 % | **621** | R82 | 11 | 0,003 % | 99,279 % | **1058** | A03 | 2 | 0,001 % | 99,919 % |
| **185** | T17 | 250 | 0,071 % | 91,122 % | **622** | R94 | 11 | 0,003 % | 99,282 % | **1059** | C88 | 2 | 0,001 % | 99,920 % |
| **186** | E10 | 250 | 0,071 % | 91,193 % | **623** | T03 | 11 | 0,003 % | 99,285 % | **1060** | D33 | 2 | 0,001 % | 99,921 % |
| **187** | H01 | 249 | 0,071 % | 91,264 % | **624** | S44 | 11 | 0,003 % | 99,288 % | **1061** | B19 | 2 | 0,001 % | 99,921 % |
| **188** | F33 | 249 | 0,071 % | 91,336 % | **625** | S97 | 11 | 0,003 % | 99,291 % | **1062** | D22 | 2 | 0,001 % | 99,922 % |
| **189** | N61 | 245 | 0,070 % | 91,406 % | **626** | M45 | 11 | 0,003 % | 99,295 % | **1063** | B83 | 2 | 0,001 % | 99,922 % |
| **190** | I82 | 245 | 0,070 % | 91,476 % | **627** | N51 | 11 | 0,003 % | 99,298 % | **1064** | A52 | 2 | 0,001 % | 99,923 % |
| **191** | W01 | 244 | 0,070 % | 91,545 % | **628** | K74 | 11 | 0,003 % | 99,301 % | **1065** | E21 | 2 | 0,001 % | 99,923 % |
| **192** | B08 | 242 | 0,069 % | 91,614 % | **629** | K75 | 11 | 0,003 % | 99,304 % | **1066** | D86 | 2 | 0,001 % | 99,924 % |
| **193** | K12 | 239 | 0,068 % | 91,683 % | **630** | L84 | 11 | 0,003 % | 99,307 % | **1067** | D26 | 2 | 0,001 % | 99,925 % |
| **194** | J40 | 227 | 0,065 % | 91,747 % | **631** | H13 | 11 | 0,003 % | 99,310 % | **1068** | D16 | 2 | 0,001 % | 99,925 % |
| **195** | M94 | 224 | 0,064 % | 91,811 % | **632** | G82 | 11 | 0,003 % | 99,313 % | **1069** | C45 | 2 | 0,001 % | 99,926 % |
| **196** | I95 | 214 | 0,061 % | 91,873 % | **633** | F95 | 11 | 0,003 % | 99,317 % | **1070** | C81 | 2 | 0,001 % | 99,926 % |
| **197** | D64 | 214 | 0,061 % | 91,934 % | **634** | I71 | 11 | 0,003 % | 99,320 % | **1071** | B15 | 2 | 0,001 % | 99,927 % |
| **198** | N12 | 212 | 0,061 % | 91,994 % | **635** | I30 | 11 | 0,003 % | 99,323 % | **1072** | C17 | 2 | 0,001 % | 99,927 % |
| **199** | M76 | 207 | 0,059 % | 92,053 % | **636** | G12 | 11 | 0,003 % | 99,326 % | **1073** | E30 | 2 | 0,001 % | 99,928 % |
| **200** | G51 | 205 | 0,059 % | 92,112 % | **637** | E04 | 11 | 0,003 % | 99,329 % | **1074** | C48 | 2 | 0,001 % | 99,929 % |
| **201** | N45 | 204 | 0,058 % | 92,170 % | **638** | C64 | 11 | 0,003 % | 99,332 % | **1075** | E71 | 2 | 0,001 % | 99,929 % |
| **202** | M43 | 203 | 0,058 % | 92,228 % | **639** | C16 | 11 | 0,003 % | 99,335 % | **1076** | C26 | 2 | 0,001 % | 99,930 % |
| **203** | T65 | 201 | 0,057 % | 92,286 % | **640** | W59 | 10 | 0,003 % | 99,338 % | **1077** | D12 | 2 | 0,001 % | 99,930 % |
| **204** | R09 | 195 | 0,056 % | 92,341 % | **641** | T62 | 10 | 0,003 % | 99,341 % | **1078** | C53 | 2 | 0,001 % | 99,931 % |
| **205** | S09 | 195 | 0,056 % | 92,397 % | **642** | Z51 | 10 | 0,003 % | 99,344 % | **1079** | E80 | 2 | 0,001 % | 99,931 % |
| **206** | R22 | 193 | 0,055 % | 92,452 % | **643** | Y19 | 10 | 0,003 % | 99,347 % | **1080** | B65 | 2 | 0,001 % | 99,932 % |
| **207** | O91 | 193 | 0,055 % | 92,507 % | **644** | T39 | 10 | 0,003 % | 99,350 % | **1081** | A15 | 2 | 0,001 % | 99,933 % |
| **208** | S29 | 191 | 0,055 % | 92,562 % | **645** | V18 | 10 | 0,003 % | 99,353 % | **1082** | E23 | 2 | 0,001 % | 99,933 % |
| **209** | Y91 | 189 | 0,054 % | 92,616 % | **646** | V48 | 10 | 0,003 % | 99,355 % | **1083** | A54 | 2 | 0,001 % | 99,934 % |
| **210** | M51 | 188 | 0,054 % | 92,669 % | **647** | Y00 | 10 | 0,003 % | 99,358 % | **1084** | E27 | 2 | 0,001 % | 99,934 % |
| **211** | N92 | 187 | 0,053 % | 92,723 % | **648** | X47 | 10 | 0,003 % | 99,361 % | **1085** | B05 | 2 | 0,001 % | 99,935 % |
| **212** | T81 | 186 | 0,053 % | 92,776 % | **649** | T35 | 10 | 0,003 % | 99,364 % | **1086** | D66 | 2 | 0,001 % | 99,935 % |
| **213** | R73 | 182 | 0,052 % | 92,828 % | **650** | P22 | 10 | 0,003 % | 99,367 % | **1087** | B49 | 2 | 0,001 % | 99,936 % |
| **214** | T18 | 182 | 0,052 % | 92,880 % | **651** | T08 | 10 | 0,003 % | 99,370 % | **1088** | C57 | 2 | 0,001 % | 99,937 % |
| **215** | B00 | 181 | 0,052 % | 92,932 % | **652** | K46 | 10 | 0,003 % | 99,373 % | **1089** | W93 | 1 | 0,000 % | 99,937 % |
| **216** | S49 | 180 | 0,051 % | 92,983 % | **653** | L42 | 10 | 0,003 % | 99,375 % | **1090** | T85 | 1 | 0,000 % | 99,937 % |
| **217** | H61 | 174 | 0,050 % | 93,033 % | **654** | K27 | 10 | 0,003 % | 99,378 % | **1091** | T53 | 1 | 0,000 % | 99,937 % |
| **218** | S31 | 169 | 0,048 % | 93,081 % | **655** | N13 | 10 | 0,003 % | 99,381 % | **1092** | Y50 | 1 | 0,000 % | 99,938 % |
| **219** | H20 | 169 | 0,048 % | 93,129 % | **656** | L24 | 10 | 0,003 % | 99,384 % | **1093** | W32 | 1 | 0,000 % | 99,938 % |
| **220** | M71 | 168 | 0,048 % | 93,177 % | **657** | G46 | 10 | 0,003 % | 99,387 % | **1094** | W20 | 1 | 0,000 % | 99,938 % |
| **221** | G30 | 168 | 0,048 % | 93,225 % | **658** | F55 | 10 | 0,003 % | 99,390 % | **1095** | T82 | 1 | 0,000 % | 99,939 % |
| **222** | F13 | 168 | 0,048 % | 93,273 % | **659** | J84 | 10 | 0,003 % | 99,393 % | **1096** | Z33 | 1 | 0,000 % | 99,939 % |
| **223** | R30 | 167 | 0,048 % | 93,321 % | **660** | F53 | 10 | 0,003 % | 99,395 % | **1097** | Z13 | 1 | 0,000 % | 99,939 % |
| **224** | T36 | 165 | 0,047 % | 93,368 % | **661** | C22 | 10 | 0,003 % | 99,398 % | **1098** | Y51 | 1 | 0,000 % | 99,939 % |
| **225** | Z73 | 163 | 0,047 % | 93,415 % | **662** | D72 | 10 | 0,003 % | 99,401 % | **1099** | W29 | 1 | 0,000 % | 99,940 % |
| **226** | F19 | 163 | 0,047 % | 93,461 % | **663** | T83 | 9 | 0,003 % | 99,404 % | **1100** | Z35 | 1 | 0,000 % | 99,940 % |
| **227** | N93 | 162 | 0,046 % | 93,507 % | **664** | T73 | 9 | 0,003 % | 99,406 % | **1101** | X82 | 1 | 0,000 % | 99,940 % |
| **228** | F22 | 162 | 0,046 % | 93,554 % | **665** | X84 | 9 | 0,003 % | 99,409 % | **1102** | Y53 | 1 | 0,000 % | 99,941 % |
| **229** | M72 | 160 | 0,046 % | 93,599 % | **666** | T96 | 9 | 0,003 % | 99,411 % | **1103** | Y98 | 1 | 0,000 % | 99,941 % |
| **230** | K61 | 159 | 0,045 % | 93,645 % | **667** | Z88 | 9 | 0,003 % | 99,414 % | **1104** | T61 | 1 | 0,000 % | 99,941 % |
| **231** | N94 | 155 | 0,044 % | 93,689 % | **668** | X20 | 9 | 0,003 % | 99,417 % | **1105** | T64 | 1 | 0,000 % | 99,941 % |
| **232** | M22 | 155 | 0,044 % | 93,733 % | **669** | X50 | 9 | 0,003 % | 99,419 % | **1106** | W03 | 1 | 0,000 % | 99,942 % |
| **233** | O21 | 154 | 0,044 % | 93,777 % | **670** | X63 | 9 | 0,003 % | 99,422 % | **1107** | Y43 | 1 | 0,000 % | 99,942 % |
| **234** | N41 | 154 | 0,044 % | 93,821 % | **671** | S84 | 9 | 0,003 % | 99,424 % | **1108** | Y58 | 1 | 0,000 % | 99,942 % |
| **235** | T79 | 151 | 0,043 % | 93,864 % | **672** | R34 | 9 | 0,003 % | 99,427 % | **1109** | X31 | 1 | 0,000 % | 99,943 % |
| **236** | E86 | 151 | 0,043 % | 93,907 % | **673** | T07 | 9 | 0,003 % | 99,429 % | **1110** | V04 | 1 | 0,000 % | 99,943 % |
| **237** | O03 | 147 | 0,042 % | 93,949 % | **674** | O05 | 9 | 0,003 % | 99,432 % | **1111** | W52 | 1 | 0,000 % | 99,943 % |
| **238** | G20 | 147 | 0,042 % | 93,991 % | **675** | K65 | 9 | 0,003 % | 99,435 % | **1112** | Y06 | 1 | 0,000 % | 99,943 % |
| **239** | T75 | 146 | 0,042 % | 94,033 % | **676** | L22 | 9 | 0,003 % | 99,437 % | **1113** | W31 | 1 | 0,000 % | 99,944 % |
| **240** | S03 | 145 | 0,041 % | 94,075 % | **677** | K82 | 9 | 0,003 % | 99,440 % | **1114** | X66 | 1 | 0,000 % | 99,944 % |
| **241** | T00 | 145 | 0,041 % | 94,116 % | **678** | L85 | 9 | 0,003 % | 99,442 % | **1115** | V39 | 1 | 0,000 % | 99,944 % |
| **242** | I25 | 144 | 0,041 % | 94,157 % | **679** | N28 | 9 | 0,003 % | 99,445 % | **1116** | X67 | 1 | 0,000 % | 99,945 % |
| **243** | R45 | 143 | 0,041 % | 94,198 % | **680** | H17 | 9 | 0,003 % | 99,447 % | **1117** | V68 | 1 | 0,000 % | 99,945 % |
| **244** | N71 | 142 | 0,041 % | 94,238 % | **681** | G00 | 9 | 0,003 % | 99,450 % | **1118** | W04 | 1 | 0,000 % | 99,945 % |
| **245** | G56 | 142 | 0,041 % | 94,279 % | **682** | J34 | 9 | 0,003 % | 99,452 % | **1119** | Z87 | 1 | 0,000 % | 99,945 % |
| **246** | D69 | 142 | 0,041 % | 94,320 % | **683** | J43 | 9 | 0,003 % | 99,455 % | **1120** | X48 | 1 | 0,000 % | 99,946 % |
| **247** | R21 | 140 | 0,040 % | 94,360 % | **684** | B07 | 9 | 0,003 % | 99,458 % | **1121** | X89 | 1 | 0,000 % | 99,946 % |
| **248** | T88 | 139 | 0,040 % | 94,399 % | **685** | B26 | 9 | 0,003 % | 99,460 % | **1122** | Z56 | 1 | 0,000 % | 99,946 % |
| **249** | S96 | 139 | 0,040 % | 94,439 % | **686** | C91 | 9 | 0,003 % | 99,463 % | **1123** | Y17 | 1 | 0,000 % | 99,947 % |
| **250** | J32 | 139 | 0,040 % | 94,479 % | **687** | Z95 | 8 | 0,002 % | 99,465 % | **1124** | X76 | 1 | 0,000 % | 99,947 % |
| **251** | B27 | 138 | 0,039 % | 94,518 % | **688** | T87 | 8 | 0,002 % | 99,467 % | **1125** | Y23 | 1 | 0,000 % | 99,947 % |
| **252** | K70 | 136 | 0,039 % | 94,557 % | **689** | X45 | 8 | 0,002 % | 99,470 % | **1126** | Z58 | 1 | 0,000 % | 99,947 % |
| **253** | F00 | 134 | 0,038 % | 94,595 % | **690** | T44 | 8 | 0,002 % | 99,472 % | **1127** | X94 | 1 | 0,000 % | 99,948 % |
| **254** | R54 | 133 | 0,038 % | 94,633 % | **691** | V28 | 8 | 0,002 % | 99,474 % | **1128** | X36 | 1 | 0,000 % | 99,948 % |
| **255** | Z01 | 131 | 0,037 % | 94,671 % | **692** | W08 | 8 | 0,002 % | 99,476 % | **1129** | Z11 | 1 | 0,000 % | 99,948 % |
| **256** | J10 | 131 | 0,037 % | 94,708 % | **693** | S54 | 8 | 0,002 % | 99,479 % | **1130** | Y89 | 1 | 0,000 % | 99,949 % |
| **257** | L29 | 130 | 0,037 % | 94,745 % | **694** | T28 | 8 | 0,002 % | 99,481 % | **1131** | X30 | 1 | 0,000 % | 99,949 % |
| **258** | T11 | 129 | 0,037 % | 94,782 % | **695** | S37 | 8 | 0,002 % | 99,483 % | **1132** | Z61 | 1 | 0,000 % | 99,949 % |
| **259** | T21 | 129 | 0,037 % | 94,819 % | **696** | T29 | 8 | 0,002 % | 99,486 % | **1133** | Z24 | 1 | 0,000 % | 99,949 % |
| **260** | K51 | 128 | 0,037 % | 94,855 % | **697** | K45 | 8 | 0,002 % | 99,488 % | **1134** | T48 | 1 | 0,000 % | 99,950 % |
| **261** | K05 | 128 | 0,037 % | 94,892 % | **698** | O04 | 8 | 0,002 % | 99,490 % | **1135** | Z26 | 1 | 0,000 % | 99,950 % |
| **262** | Z76 | 127 | 0,036 % | 94,928 % | **699** | K91 | 8 | 0,002 % | 99,492 % | **1136** | T71 | 1 | 0,000 % | 99,950 % |
| **263** | K86 | 126 | 0,036 % | 94,964 % | **700** | M32 | 8 | 0,002 % | 99,495 % | **1137** | W69 | 1 | 0,000 % | 99,951 % |
| **264** | K81 | 126 | 0,036 % | 95,000 % | **701** | L25 | 8 | 0,002 % | 99,497 % | **1138** | Z70 | 1 | 0,000 % | 99,951 % |
| **265** | S41 | 124 | 0,035 % | 95,036 % | **702** | M86 | 8 | 0,002 % | 99,499 % | **1139** | Q01 | 1 | 0,000 % | 99,951 % |
| **266** | W45 | 123 | 0,035 % | 95,071 % | **703** | M08 | 8 | 0,002 % | 99,502 % | **1140** | O75 | 1 | 0,000 % | 99,951 % |
| **267** | H73 | 122 | 0,035 % | 95,106 % | **704** | L56 | 8 | 0,002 % | 99,504 % | **1141** | Q99 | 1 | 0,000 % | 99,952 % |
| **268** | J09 | 120 | 0,034 % | 95,140 % | **705** | F98 | 8 | 0,002 % | 99,506 % | **1142** | Q32 | 1 | 0,000 % | 99,952 % |
| **269** | L23 | 116 | 0,033 % | 95,173 % | **706** | I22 | 8 | 0,002 % | 99,508 % | **1143** | O67 | 1 | 0,000 % | 99,952 % |
| **270** | T24 | 114 | 0,033 % | 95,206 % | **707** | K01 | 8 | 0,002 % | 99,511 % | **1144** | Q38 | 1 | 0,000 % | 99,953 % |
| **271** | F99 | 113 | 0,032 % | 95,238 % | **708** | K06 | 8 | 0,002 % | 99,513 % | **1145** | T27 | 1 | 0,000 % | 99,953 % |
| **272** | Y90 | 111 | 0,032 % | 95,270 % | **709** | C67 | 8 | 0,002 % | 99,515 % | **1146** | Q39 | 1 | 0,000 % | 99,953 % |
| **273** | M24 | 108 | 0,031 % | 95,300 % | **710** | D70 | 8 | 0,002 % | 99,518 % | **1147** | Q23 | 1 | 0,000 % | 99,953 % |
| **274** | K58 | 107 | 0,031 % | 95,331 % | **711** | E83 | 8 | 0,002 % | 99,520 % | **1148** | S47 | 1 | 0,000 % | 99,954 % |
| **275** | D50 | 107 | 0,031 % | 95,361 % | **712** | A74 | 8 | 0,002 % | 99,522 % | **1149** | O92 | 1 | 0,000 % | 99,954 % |
| **276** | N77 | 104 | 0,030 % | 95,391 % | **713** | D37 | 8 | 0,002 % | 99,524 % | **1150** | S95 | 1 | 0,000 % | 99,954 % |
| **277** | B35 | 103 | 0,029 % | 95,421 % | **714** | T84 | 7 | 0,002 % | 99,526 % | **1151** | Q79 | 1 | 0,000 % | 99,955 % |
| **278** | N73 | 101 | 0,029 % | 95,449 % | **715** | Z41 | 7 | 0,002 % | 99,528 % | **1152** | O68 | 1 | 0,000 % | 99,955 % |
| **279** | F60 | 101 | 0,029 % | 95,478 % | **716** | ZA5 | 7 | 0,002 % | 99,530 % | **1153** | P20 | 1 | 0,000 % | 99,955 % |
| **280** | T20 | 100 | 0,029 % | 95,507 % | **717** | X62 | 7 | 0,002 % | 99,532 % | **1154** | O87 | 1 | 0,000 % | 99,955 % |
| **281** | S79 | 99 | 0,028 % | 95,535 % | **718** | Z59 | 7 | 0,002 % | 99,534 % | **1155** | Q96 | 1 | 0,000 % | 99,956 % |
| **282** | R23 | 98 | 0,028 % | 95,563 % | **719** | W11 | 7 | 0,002 % | 99,536 % | **1156** | S98 | 1 | 0,000 % | 99,956 % |
| **283** | I83 | 98 | 0,028 % | 95,591 % | **720** | ZA2 | 7 | 0,002 % | 99,538 % | **1157** | O85 | 1 | 0,000 % | 99,956 % |
| **284** | F25 | 97 | 0,028 % | 95,619 % | **721** | W18 | 7 | 0,002 % | 99,540 % | **1158** | Q50 | 1 | 0,000 % | 99,957 % |
| **285** | M16 | 96 | 0,027 % | 95,646 % | **722** | T46 | 7 | 0,002 % | 99,542 % | **1159** | Q71 | 1 | 0,000 % | 99,957 % |
| **286** | H83 | 95 | 0,027 % | 95,673 % | **723** | Y59 | 7 | 0,002 % | 99,544 % | **1160** | Q52 | 1 | 0,000 % | 99,957 % |
| **287** | T63 | 93 | 0,027 % | 95,700 % | **724** | R69 | 7 | 0,002 % | 99,546 % | **1161** | R70 | 1 | 0,000 % | 99,957 % |
| **288** | T13 | 93 | 0,027 % | 95,726 % | **725** | O86 | 7 | 0,002 % | 99,548 % | **1162** | Q53 | 1 | 0,000 % | 99,958 % |
| **289** | S73 | 93 | 0,027 % | 95,753 % | **726** | T06 | 7 | 0,002 % | 99,550 % | **1163** | S26 | 1 | 0,000 % | 99,958 % |
| **290** | L60 | 93 | 0,027 % | 95,780 % | **727** | N36 | 7 | 0,002 % | 99,552 % | **1164** | P38 | 1 | 0,000 % | 99,958 % |
| **291** | I74 | 93 | 0,027 % | 95,806 % | **728** | M11 | 7 | 0,002 % | 99,554 % | **1165** | P03 | 1 | 0,000 % | 99,959 % |
| **292** | I26 | 92 | 0,026 % | 95,832 % | **729** | L51 | 7 | 0,002 % | 99,556 % | **1166** | O88 | 1 | 0,000 % | 99,959 % |
| **293** | F40 | 92 | 0,026 % | 95,859 % | **730** | L55 | 7 | 0,002 % | 99,558 % | **1167** | Q80 | 1 | 0,000 % | 99,959 % |
| **294** | Y57 | 91 | 0,026 % | 95,885 % | **731** | O14 | 7 | 0,002 % | 99,560 % | **1168** | R15 | 1 | 0,000 % | 99,959 % |
| **295** | R17 | 91 | 0,026 % | 95,911 % | **732** | M31 | 7 | 0,002 % | 99,562 % | **1169** | Q07 | 1 | 0,000 % | 99,960 % |
| **296** | S10 | 86 | 0,025 % | 95,935 % | **733** | N72 | 7 | 0,002 % | 99,564 % | **1170** | Q63 | 1 | 0,000 % | 99,960 % |
| **297** | M48 | 86 | 0,025 % | 95,960 % | **734** | H35 | 7 | 0,002 % | 99,566 % | **1171** | R89 | 1 | 0,000 % | 99,960 % |
| **298** | F34 | 86 | 0,025 % | 95,984 % | **735** | J13 | 7 | 0,002 % | 99,568 % | **1172** | Q64 | 1 | 0,000 % | 99,961 % |
| **299** | R68 | 85 | 0,024 % | 96,009 % | **736** | G80 | 7 | 0,002 % | 99,570 % | **1173** | Q90 | 1 | 0,000 % | 99,961 % |
| **300** | R35 | 85 | 0,024 % | 96,033 % | **737** | H21 | 7 | 0,002 % | 99,572 % | **1174** | O64 | 1 | 0,000 % | 99,961 % |
| **301** | L27 | 85 | 0,024 % | 96,057 % | **738** | H69 | 7 | 0,002 % | 99,574 % | **1175** | R93 | 1 | 0,000 % | 99,961 % |
| **302** | G35 | 85 | 0,024 % | 96,082 % | **739** | F70 | 7 | 0,002 % | 99,576 % | **1176** | Q66 | 1 | 0,000 % | 99,962 % |
| **303** | K42 | 82 | 0,023 % | 96,105 % | **740** | I34 | 7 | 0,002 % | 99,578 % | **1177** | T32 | 1 | 0,000 % | 99,962 % |
| **304** | F30 | 82 | 0,023 % | 96,128 % | **741** | I35 | 7 | 0,002 % | 99,580 % | **1178** | S18 | 1 | 0,000 % | 99,962 % |
| **305** | T16 | 81 | 0,023 % | 96,151 % | **742** | I86 | 7 | 0,002 % | 99,582 % | **1179** | Q22 | 1 | 0,000 % | 99,963 % |
| **306** | K37 | 79 | 0,023 % | 96,174 % | **743** | H44 | 7 | 0,002 % | 99,584 % | **1180** | Q68 | 1 | 0,000 % | 99,963 % |
| **307** | B37 | 79 | 0,023 % | 96,197 % | **744** | J47 | 7 | 0,002 % | 99,586 % | **1181** | Q25 | 1 | 0,000 % | 99,963 % |
| **308** | I64 | 78 | 0,022 % | 96,219 % | **745** | A21 | 7 | 0,002 % | 99,588 % | **1182** | Q69 | 1 | 0,000 % | 99,963 % |
| **309** | F03 | 77 | 0,022 % | 96,241 % | **746** | A48 | 7 | 0,002 % | 99,590 % | **1183** | L66 | 1 | 0,000 % | 99,964 % |
| **310** | R40 | 76 | 0,022 % | 96,263 % | **747** | E29 | 7 | 0,002 % | 99,592 % | **1184** | O42 | 1 | 0,000 % | 99,964 % |
| **311** | S33 | 76 | 0,022 % | 96,284 % | **748** | D38 | 7 | 0,002 % | 99,594 % | **1185** | K73 | 1 | 0,000 % | 99,964 % |
| **312** | F15 | 76 | 0,022 % | 96,306 % | **749** | C56 | 7 | 0,002 % | 99,596 % | **1186** | N98 | 1 | 0,000 % | 99,965 % |
| **313** | T25 | 75 | 0,021 % | 96,327 % | **750** | F07 | 7 | 0,002 % | 99,598 % | **1187** | N03 | 1 | 0,000 % | 99,965 % |
| **314** | N44 | 75 | 0,021 % | 96,349 % | **751** | C20 | 7 | 0,002 % | 99,600 % | **1188** | M01 | 1 | 0,000 % | 99,965 % |
| **315** | I61 | 75 | 0,021 % | 96,370 % | **752** | D32 | 7 | 0,002 % | 99,602 % | **1189** | L26 | 1 | 0,000 % | 99,965 % |
| **316** | R13 | 74 | 0,021 % | 96,391 % | **753** | Z57 | 6 | 0,002 % | 99,604 % | **1190** | N74 | 1 | 0,000 % | 99,966 % |
| **317** | Y94 | 73 | 0,021 % | 96,412 % | **754** | Z22 | 6 | 0,002 % | 99,606 % | **1191** | O28 | 1 | 0,000 % | 99,966 % |
| **318** | R58 | 72 | 0,021 % | 96,433 % | **755** | ZA3 | 6 | 0,002 % | 99,608 % | **1192** | N26 | 1 | 0,000 % | 99,966 % |
| **319** | O47 | 72 | 0,021 % | 96,453 % | **756** | X60 | 6 | 0,002 % | 99,609 % | **1193** | N97 | 1 | 0,000 % | 99,967 % |
| **320** | L04 | 71 | 0,020 % | 96,474 % | **757** | Z50 | 6 | 0,002 % | 99,611 % | **1194** | L12 | 1 | 0,000 % | 99,967 % |
| **321** | H43 | 71 | 0,020 % | 96,494 % | **758** | Y14 | 6 | 0,002 % | 99,613 % | **1195** | L80 | 1 | 0,000 % | 99,967 % |
| **322** | B43 | 71 | 0,020 % | 96,514 % | **759** | W46 | 6 | 0,002 % | 99,614 % | **1196** | L82 | 1 | 0,000 % | 99,967 % |
| **323** | M00 | 70 | 0,020 % | 96,534 % | **760** | Y32 | 6 | 0,002 % | 99,616 % | **1197** | L94 | 1 | 0,000 % | 99,968 % |
| **324** | K60 | 70 | 0,020 % | 96,554 % | **761** | S94 | 6 | 0,002 % | 99,618 % | **1198** | M95 | 1 | 0,000 % | 99,968 % |
| **325** | I87 | 70 | 0,020 % | 96,574 % | **762** | O72 | 6 | 0,002 % | 99,620 % | **1199** | M73 | 1 | 0,000 % | 99,968 % |
| **326** | T42 | 69 | 0,020 % | 96,594 % | **763** | Q82 | 6 | 0,002 % | 99,621 % | **1200** | M12 | 1 | 0,000 % | 99,969 % |
| **327** | K50 | 69 | 0,020 % | 96,614 % | **764** | S36 | 6 | 0,002 % | 99,623 % | **1201** | M68 | 1 | 0,000 % | 99,969 % |
| **328** | G72 | 69 | 0,020 % | 96,633 % | **765** | O60 | 6 | 0,002 % | 99,625 % | **1202** | L64 | 1 | 0,000 % | 99,969 % |
| **329** | F11 | 69 | 0,020 % | 96,653 % | **766** | T10 | 6 | 0,002 % | 99,626 % | **1203** | N01 | 1 | 0,000 % | 99,969 % |
| **330** | Z32 | 68 | 0,019 % | 96,672 % | **767** | O02 | 6 | 0,002 % | 99,628 % | **1204** | M49 | 1 | 0,000 % | 99,970 % |
| **331** | T26 | 68 | 0,019 % | 96,692 % | **768** | K26 | 6 | 0,002 % | 99,630 % | **1205** | N96 | 1 | 0,000 % | 99,970 % |
| **332** | M06 | 68 | 0,019 % | 96,711 % | **769** | L28 | 6 | 0,002 % | 99,632 % | **1206** | M42 | 1 | 0,000 % | 99,970 % |
| **333** | K08 | 68 | 0,019 % | 96,731 % | **770** | O23 | 6 | 0,002 % | 99,633 % | **1207** | L65 | 1 | 0,000 % | 99,971 % |
| **334** | Z63 | 67 | 0,019 % | 96,750 % | **771** | L92 | 6 | 0,002 % | 99,635 % | **1208** | I05 | 1 | 0,000 % | 99,971 % |
| **335** | T22 | 67 | 0,019 % | 96,769 % | **772** | N89 | 6 | 0,002 % | 99,637 % | **1209** | K03 | 1 | 0,000 % | 99,971 % |
| **336** | G81 | 67 | 0,019 % | 96,788 % | **773** | M93 | 6 | 0,002 % | 99,638 % | **1210** | F64 | 1 | 0,000 % | 99,971 % |
| **337** | G24 | 67 | 0,019 % | 96,807 % | **774** | M92 | 6 | 0,002 % | 99,640 % | **1211** | F78 | 1 | 0,000 % | 99,972 % |
| **338** | M67 | 66 | 0,019 % | 96,826 % | **775** | N60 | 6 | 0,002 % | 99,642 % | **1212** | H48 | 1 | 0,000 % | 99,972 % |
| **339** | G50 | 66 | 0,019 % | 96,845 % | **776** | M14 | 6 | 0,002 % | 99,644 % | **1213** | G37 | 1 | 0,000 % | 99,972 % |
| **340** | B30 | 66 | 0,019 % | 96,864 % | **777** | L43 | 6 | 0,002 % | 99,645 % | **1214** | J95 | 1 | 0,000 % | 99,973 % |
| **341** | F39 | 66 | 0,019 % | 96,883 % | **778** | H26 | 6 | 0,002 % | 99,647 % | **1215** | J14 | 1 | 0,000 % | 99,973 % |
| **342** | A69 | 66 | 0,019 % | 96,901 % | **779** | G71 | 6 | 0,002 % | 99,649 % | **1216** | H58 | 1 | 0,000 % | 99,973 % |
| **343** | L40 | 65 | 0,019 % | 96,920 % | **780** | H67 | 6 | 0,002 % | 99,650 % | **1217** | J67 | 1 | 0,000 % | 99,973 % |
| **344** | N17 | 65 | 0,019 % | 96,939 % | **781** | I62 | 6 | 0,002 % | 99,652 % | **1218** | F52 | 1 | 0,000 % | 99,974 % |
| **345** | H04 | 65 | 0,019 % | 96,957 % | **782** | J68 | 6 | 0,002 % | 99,654 % | **1219** | H27 | 1 | 0,000 % | 99,974 % |
| **346** | A04 | 65 | 0,019 % | 96,976 % | **783** | E78 | 6 | 0,002 % | 99,656 % | **1220** | I85 | 1 | 0,000 % | 99,974 % |
| **347** | Z91 | 63 | 0,018 % | 96,994 % | **784** | B54 | 6 | 0,002 % | 99,657 % | **1221** | H51 | 1 | 0,000 % | 99,975 % |
| **348** | G47 | 62 | 0,018 % | 97,011 % | **785** | A02 | 6 | 0,002 % | 99,659 % | **1222** | G06 | 1 | 0,000 % | 99,975 % |
| **349** | A05 | 62 | 0,018 % | 97,029 % | **786** | C43 | 6 | 0,002 % | 99,661 % | **1223** | H31 | 1 | 0,000 % | 99,975 % |
| **350** | F21 | 62 | 0,018 % | 97,047 % | **787** | D48 | 6 | 0,002 % | 99,662 % | **1224** | I79 | 1 | 0,000 % | 99,975 % |
| **351** | R18 | 61 | 0,017 % | 97,064 % | **788** | B97 | 6 | 0,002 % | 99,664 % | **1225** | H06 | 1 | 0,000 % | 99,976 % |
| **352** | N40 | 61 | 0,017 % | 97,082 % | **789** | C80 | 6 | 0,002 % | 99,666 % | **1226** | F54 | 1 | 0,000 % | 99,976 % |
| **353** | N75 | 61 | 0,017 % | 97,099 % | **790** | C79 | 6 | 0,002 % | 99,668 % | **1227** | J82 | 1 | 0,000 % | 99,976 % |
| **354** | G57 | 61 | 0,017 % | 97,117 % | **791** | A36 | 6 | 0,002 % | 99,669 % | **1228** | F81 | 1 | 0,000 % | 99,977 % |
| **355** | B99 | 61 | 0,017 % | 97,134 % | **792** | D40 | 6 | 0,002 % | 99,671 % | **1229** | I27 | 1 | 0,000 % | 99,977 % |
| **356** | S21 | 60 | 0,017 % | 97,151 % | **793** | E51 | 6 | 0,002 % | 99,673 % | **1230** | I12 | 1 | 0,000 % | 99,977 % |
| **357** | T51 | 59 | 0,017 % | 97,168 % | **794** | C78 | 6 | 0,002 % | 99,674 % | **1231** | G99 | 1 | 0,000 % | 99,977 % |
| **358** | Z43 | 59 | 0,017 % | 97,185 % | **795** | Z82 | 5 | 0,001 % | 99,676 % | **1232** | H47 | 1 | 0,000 % | 99,978 % |
| **359** | I70 | 59 | 0,017 % | 97,202 % | **796** | V69 | 5 | 0,001 % | 99,677 % | **1233** | I98 | 1 | 0,000 % | 99,978 % |
| **360** | S59 | 58 | 0,017 % | 97,218 % | **797** | W06 | 5 | 0,001 % | 99,679 % | **1234** | G92 | 1 | 0,000 % | 99,978 % |
| **361** | L53 | 58 | 0,017 % | 97,235 % | **798** | Y86 | 5 | 0,001 % | 99,680 % | **1235** | I00 | 1 | 0,000 % | 99,979 % |
| **362** | F91 | 58 | 0,017 % | 97,251 % | **799** | W78 | 5 | 0,001 % | 99,682 % | **1236** | J91 | 1 | 0,000 % | 99,979 % |
| **363** | Y04 | 57 | 0,016 % | 97,268 % | **800** | W84 | 5 | 0,001 % | 99,683 % | **1237** | F69 | 1 | 0,000 % | 99,979 % |
| **364** | H72 | 57 | 0,016 % | 97,284 % | **801** | V79 | 5 | 0,001 % | 99,684 % | **1238** | J65 | 1 | 0,000 % | 99,979 % |
| **365** | ZA1 | 56 | 0,016 % | 97,300 % | **802** | V14 | 5 | 0,001 % | 99,686 % | **1239** | D75 | 1 | 0,000 % | 99,980 % |
| **366** | R39 | 56 | 0,016 % | 97,316 % | **803** | V09 | 5 | 0,001 % | 99,687 % | **1240** | C19 | 1 | 0,000 % | 99,980 % |
| **367** | Z71 | 55 | 0,016 % | 97,332 % | **804** | V01 | 5 | 0,001 % | 99,689 % | **1241** | C06 | 1 | 0,000 % | 99,980 % |
| **368** | K20 | 55 | 0,016 % | 97,347 % | **805** | O99 | 5 | 0,001 % | 99,690 % | **1242** | D06 | 1 | 0,000 % | 99,981 % |
| **369** | N19 | 54 | 0,015 % | 97,363 % | **806** | R43 | 5 | 0,001 % | 99,692 % | **1243** | D24 | 1 | 0,000 % | 99,981 % |
| **370** | O00 | 53 | 0,015 % | 97,378 % | **807** | R91 | 5 | 0,001 % | 99,693 % | **1244** | A77 | 1 | 0,000 % | 99,981 % |
| **371** | I89 | 53 | 0,015 % | 97,393 % | **808** | O62 | 5 | 0,001 % | 99,694 % | **1245** | D01 | 1 | 0,000 % | 99,981 % |
| **372** | J93 | 53 | 0,015 % | 97,408 % | **809** | S14 | 5 | 0,001 % | 99,696 % | **1246** | B81 | 1 | 0,000 % | 99,982 % |
| **373** | J31 | 53 | 0,015 % | 97,423 % | **810** | S34 | 5 | 0,001 % | 99,697 % | **1247** | D56 | 1 | 0,000 % | 99,982 % |
| **374** | A38 | 53 | 0,015 % | 97,438 % | **811** | R98 | 5 | 0,001 % | 99,699 % | **1248** | B82 | 1 | 0,000 % | 99,982 % |
| **375** | F45 | 53 | 0,015 % | 97,454 % | **812** | S08 | 5 | 0,001 % | 99,700 % | **1249** | A16 | 1 | 0,000 % | 99,983 % |
| **376** | Z30 | 52 | 0,015 % | 97,468 % | **813** | S07 | 5 | 0,001 % | 99,702 % | **1250** | D07 | 1 | 0,000 % | 99,983 % |
| **377** | Z09 | 52 | 0,015 % | 97,483 % | **814** | L74 | 5 | 0,001 % | 99,703 % | **1251** | B50 | 1 | 0,000 % | 99,983 % |
| **378** | V29 | 52 | 0,015 % | 97,498 % | **815** | N42 | 5 | 0,001 % | 99,704 % | **1252** | E15 | 1 | 0,000 % | 99,983 % |
| **379** | R49 | 52 | 0,015 % | 97,513 % | **816** | K41 | 5 | 0,001 % | 99,706 % | **1253** | D00 | 1 | 0,000 % | 99,984 % |
| **380** | S12 | 51 | 0,015 % | 97,528 % | **817** | M18 | 5 | 0,001 % | 99,707 % | **1254** | B85 | 1 | 0,000 % | 99,984 % |
| **381** | J42 | 51 | 0,015 % | 97,542 % | **818** | N63 | 5 | 0,001 % | 99,709 % | **1255** | C04 | 1 | 0,000 % | 99,984 % |
| **382** | B86 | 50 | 0,014 % | 97,556 % | **819** | G11 | 5 | 0,001 % | 99,710 % | **1256** | A59 | 1 | 0,000 % | 99,985 % |
| **383** | ZA6 | 49 | 0,014 % | 97,570 % | **820** | J33 | 5 | 0,001 % | 99,712 % | **1257** | A01 | 1 | 0,000 % | 99,985 % |
| **384** | M66 | 49 | 0,014 % | 97,584 % | **821** | J86 | 5 | 0,001 % | 99,713 % | **1258** | E22 | 1 | 0,000 % | 99,985 % |
| **385** | H05 | 49 | 0,014 % | 97,598 % | **822** | G23 | 5 | 0,001 % | 99,714 % | **1259** | D58 | 1 | 0,000 % | 99,985 % |
| **386** | X61 | 48 | 0,014 % | 97,612 % | **823** | G63 | 5 | 0,001 % | 99,716 % | **1260** | D39 | 1 | 0,000 % | 99,986 % |
| **387** | L05 | 48 | 0,014 % | 97,626 % | **824** | I77 | 5 | 0,001 % | 99,717 % | **1261** | B24 | 1 | 0,000 % | 99,986 % |
| **388** | H93 | 48 | 0,014 % | 97,639 % | **825** | I78 | 5 | 0,001 % | 99,719 % | **1262** | E26 | 1 | 0,000 % | 99,986 % |
| **389** | I15 | 47 | 0,013 % | 97,653 % | **826** | H34 | 5 | 0,001 % | 99,720 % | **1263** | C24 | 1 | 0,000 % | 99,987 % |
| **390** | G83 | 47 | 0,013 % | 97,666 % | **827** | H90 | 5 | 0,001 % | 99,722 % | **1264** | A30 | 1 | 0,000 % | 99,987 % |
| **391** | N21 | 46 | 0,013 % | 97,679 % | **828** | G97 | 5 | 0,001 % | 99,723 % | **1265** | B48 | 1 | 0,000 % | 99,987 % |
| **392** | C61 | 46 | 0,013 % | 97,693 % | **829** | J80 | 5 | 0,001 % | 99,724 % | **1266** | D41 | 1 | 0,000 % | 99,987 % |
| **393** | F12 | 46 | 0,013 % | 97,706 % | **830** | H49 | 5 | 0,001 % | 99,726 % | **1267** | A07 | 1 | 0,000 % | 99,988 % |
| **394** | S39 | 45 | 0,013 % | 97,719 % | **831** | F18 | 5 | 0,001 % | 99,727 % | **1268** | A81 | 1 | 0,000 % | 99,988 % |
| **395** | S56 | 45 | 0,013 % | 97,731 % | **832** | A88 | 5 | 0,001 % | 99,729 % | **1269** | E01 | 1 | 0,000 % | 99,988 % |
| **396** | N47 | 45 | 0,013 % | 97,744 % | **833** | C49 | 5 | 0,001 % | 99,730 % | **1270** | D42 | 1 | 0,000 % | 99,989 % |
| **397** | A41 | 45 | 0,013 % | 97,757 % | **834** | B18 | 5 | 0,001 % | 99,732 % | **1271** | C52 | 1 | 0,000 % | 99,989 % |
| **398** | M50 | 44 | 0,013 % | 97,770 % | **835** | C15 | 5 | 0,001 % | 99,733 % | **1272** | E34 | 1 | 0,000 % | 99,989 % |
| **399** | N23 | 44 | 0,013 % | 97,782 % | **836** | C83 | 5 | 0,001 % | 99,734 % | **1273** | A53 | 1 | 0,000 % | 99,989 % |
| **400** | M15 | 43 | 0,012 % | 97,795 % | **837** | D18 | 5 | 0,001 % | 99,736 % | **1274** | E40 | 1 | 0,000 % | 99,990 % |
| **401** | T40 | 42 | 0,012 % | 97,807 % | **838** | D47 | 5 | 0,001 % | 99,737 % | **1275** | C05 | 1 | 0,000 % | 99,990 % |
| **402** | T43 | 42 | 0,012 % | 97,819 % | **839** | X32 | 4 | 0,001 % | 99,738 % | **1276** | E41 | 1 | 0,000 % | 99,990 % |
| **403** | W17 | 42 | 0,012 % | 97,831 % | **840** | Z46 | 4 | 0,001 % | 99,740 % | **1277** | C08 | 1 | 0,000 % | 99,991 % |
| **404** | K43 | 42 | 0,012 % | 97,843 % | **841** | X70 | 4 | 0,001 % | 99,741 % | **1278** | E46 | 1 | 0,000 % | 99,991 % |
| **405** | M19 | 42 | 0,012 % | 97,855 % | **842** | T91 | 4 | 0,001 % | 99,742 % | **1279** | C10 | 1 | 0,000 % | 99,991 % |
| **406** | M60 | 42 | 0,012 % | 97,867 % | **843** | Z53 | 4 | 0,001 % | 99,743 % | **1280** | A86 | 1 | 0,000 % | 99,991 % |
| **407** | J16 | 42 | 0,012 % | 97,879 % | **844** | Y05 | 4 | 0,001 % | 99,744 % | **1281** | A90 | 1 | 0,000 % | 99,992 % |
| **408** | C34 | 42 | 0,012 % | 97,891 % | **845** | Z96 | 4 | 0,001 % | 99,745 % | **1282** | E53 | 1 | 0,000 % | 99,992 % |
| **409** | T67 | 41 | 0,012 % | 97,902 % | **846** | Y44 | 4 | 0,001 % | 99,746 % | **1283** | D59 | 1 | 0,000 % | 99,992 % |
| **410** | M05 | 41 | 0,012 % | 97,914 % | **847** | V59 | 4 | 0,001 % | 99,748 % | **1284** | E63 | 1 | 0,000 % | 99,993 % |
| **411** | K13 | 41 | 0,012 % | 97,926 % | **848** | X43 | 4 | 0,001 % | 99,749 % | **1285** | B20 | 1 | 0,000 % | 99,993 % |
| **412** | K04 | 41 | 0,012 % | 97,937 % | **849** | W25 | 4 | 0,001 % | 99,750 % | **1286** | B90 | 1 | 0,000 % | 99,993 % |
| **413** | F42 | 41 | 0,012 % | 97,949 % | **850** | T69 | 4 | 0,001 % | 99,751 % | **1287** | A93 | 1 | 0,000 % | 99,993 % |
| **414** | C50 | 40 | 0,011 % | 97,960 % | **851** | W79 | 4 | 0,001 % | 99,752 % | **1288** | C74 | 1 | 0,000 % | 99,994 % |
| **415** | A60 | 40 | 0,011 % | 97,972 % | **852** | X08 | 4 | 0,001 % | 99,753 % | **1289** | C23 | 1 | 0,000 % | 99,994 % |
| **416** | M02 | 39 | 0,011 % | 97,983 % | **853** | T52 | 4 | 0,001 % | 99,754 % | **1290** | E72 | 1 | 0,000 % | 99,994 % |
| **417** | H54 | 39 | 0,011 % | 97,994 % | **854** | ZA4 | 4 | 0,001 % | 99,756 % | **1291** | A43 | 1 | 0,000 % | 99,995 % |
| **418** | I60 | 39 | 0,011 % | 98,005 % | **855** | Z97 | 4 | 0,001 % | 99,757 % | **1292** | D44 | 1 | 0,000 % | 99,995 % |
| **419** | N34 | 38 | 0,011 % | 98,016 % | **856** | X14 | 4 | 0,001 % | 99,758 % | **1293** | C31 | 1 | 0,000 % | 99,995 % |
| **420** | J05 | 38 | 0,011 % | 98,027 % | **857** | T38 | 4 | 0,001 % | 99,759 % | **1294** | B91 | 1 | 0,000 % | 99,995 % |
| **421** | I88 | 38 | 0,011 % | 98,038 % | **858** | R64 | 4 | 0,001 % | 99,760 % | **1295** | A26 | 1 | 0,000 % | 99,996 % |
| **422** | F01 | 38 | 0,011 % | 98,049 % | **859** | R79 | 4 | 0,001 % | 99,761 % | **1296** | E79 | 1 | 0,000 % | 99,996 % |
| **423** | R59 | 37 | 0,011 % | 98,059 % | **860** | R74 | 4 | 0,001 % | 99,762 % | **1297** | B52 | 1 | 0,000 % | 99,996 % |
| **424** | S67 | 37 | 0,011 % | 98,070 % | **861** | Q73 | 4 | 0,001 % | 99,764 % | **1298** | D11 | 1 | 0,000 % | 99,997 % |
| **425** | T09 | 37 | 0,011 % | 98,080 % | **862** | R77 | 4 | 0,001 % | 99,765 % | **1299** | C44 | 1 | 0,000 % | 99,997 % |
| **426** | K22 | 37 | 0,011 % | 98,091 % | **863** | P28 | 4 | 0,001 % | 99,766 % | **1300** | B95 | 1 | 0,000 % | 99,997 % |
| **427** | L72 | 37 | 0,011 % | 98,102 % | **864** | R61 | 4 | 0,001 % | 99,767 % | **1301** | C62 | 1 | 0,000 % | 99,997 % |
| **428** | J90 | 37 | 0,011 % | 98,112 % | **865** | O80 | 4 | 0,001 % | 99,768 % | **1302** | E84 | 1 | 0,000 % | 99,998 % |
| **429** | E14 | 37 | 0,011 % | 98,123 % | **866** | P74 | 4 | 0,001 % | 99,769 % | **1303** | A75 | 1 | 0,000 % | 99,998 % |
| **430** | J94 | 36 | 0,010 % | 98,133 % | **867** | L95 | 4 | 0,001 % | 99,770 % | **1304** | B96 | 1 | 0,000 % | 99,998 % |
| **431** | S27 | 35 | 0,010 % | 98,143 % | **868** | K38 | 4 | 0,001 % | 99,772 % | **1305** | C51 | 1 | 0,000 % | 99,999 % |
| **432** | N70 | 35 | 0,010 % | 98,153 % | **869** | M36 | 4 | 0,001 % | 99,773 % | **1306** | A51 | 1 | 0,000 % | 99,999 % |
| **433** | O06 | 35 | 0,010 % | 98,163 % | **870** | M99 | 4 | 0,001 % | 99,774 % | **1307** | B76 | 1 | 0,000 % | 99,999 % |
| **434** | F48 | 35 | 0,010 % | 98,173 % | **871** | N31 | 4 | 0,001 % | 99,775 % | **1308** | D14 | 1 | 0,000 % | 99,999 % |
| **435** | T93 | 34 | 0,010 % | 98,183 % | **872** | N08 | 4 | 0,001 % | 99,776 % | **1309** | C84 | 1 | 0,000 % | 100,000 % |
| **436** | T68 | 33 | 0,009 % | 98,192 % | **873** | O12 | 4 | 0,001 % | 99,777 % | **1310** | E07 | 1 | 0,000 % | 100,000 % |
| **437** | R26 | 33 | 0,009 % | 98,202 % | **874** | M07 | 4 | 0,001 % | 99,778 % |  |  |  |  |  |
